# Supplementary material for: A subnational socioeconomic assessment of family planning levels, projections, and disparities among married women of reproductive age in Cameroon
Source: PLoS One. 2025 Feb 14;20(2):e0318650. doi: 10.1371/journal.pone.0318650 (PMC11828404; doi:10.1371/journal.pone.0318650)
Supplement: S1 Table — DHS = Demographic and Health Survey; mCPR = modern contraceptive prevalence; mUNFP = unmet need for modern methods; mDSFP = demand satisfied with modern methods. Sample = women aged 15 to 49 years who are married or in a union. (DOCX) [file pone.0318650.s001.docx]

**S1 Table: Characteristics of data used to assess family planning indicators by area of residence, wealth, and educational status across regions of Cameroon**

| Data source | Year | Sample size |  | Number of Observations | | |
| --- | --- | --- | --- | --- | --- | --- |
|  |  |  | **Area of residence** | **Family Planning Indicator** | | |
|  |  |  |  | mCPR | mUNFP | mDSFP |
| Demographic and Health Survey | 1991 | 3871 | Urban | 10 | 10 | 10 |
|  |  |  | Rural | 11 | 11 | 11 |
|  | 1998 | 5501 | Urban | 11 | 11 | 11 |
|  |  |  | Rural | 11 | 11 | 11 |
|  | 2004 | 10656 | Urban | 11 | 11 | 11 |
|  |  |  | Rural | 11 | 11 | 11 |
|  | 2011 | 15426 | Urban | 11 | 11 | 11 |
|  |  |  | Rural | 11 | 11 | 11 |
|  | 2018 | 13500 | Urban | 11 | 11 | 11 |
|  |  |  | Rural | 10 | 10 | 10 |
|  |  |  | **Wealth Quintile** |  |  |  |
|  | 1991 | 3871 | Poorest | 55 | 55 | 55 |
|  |  |  | Poorer | 55 | 55 | 55 |
|  |  |  | Middle | 55 | 55 | 55 |
|  |  |  | Richer | 55 | 55 | 55 |
|  |  |  | Richest | 55 | 55 | 55 |
|  | 1998 | 5501 | Poorest | 55 | 55 | 55 |
|  |  |  | Poorer | 55 | 55 | 55 |
|  |  |  | Middle | 55 | 55 | 55 |
|  |  |  | Richer | 55 | 55 | 55 |
|  |  |  | Richest | 55 | 55 | 55 |
|  | 2004 | 10656 | Poorest | 55 | 55 | 55 |
|  |  |  | Poorer | 55 | 55 | 55 |
|  |  |  | Middle | 55 | 55 | 55 |
|  |  |  | Richer | 55 | 55 | 55 |
|  |  |  | Richest | 55 | 55 | 55 |
|  | 2011 | 15426 | Poorest | 55 | 55 | 55 |
|  |  |  | Poorer | 55 | 55 | 55 |
|  |  |  | Middle | 55 | 55 | 55 |
|  |  |  | Richer | 55 | 55 | 55 |
|  |  |  | Richest | 55 | 55 | 55 |
|  | 2018 | 13500 | Poorest | 54 | 54 | 54 |
|  |  |  | Poorer | 54 | 54 | 54 |
|  |  |  | Middle | 55 | 55 | 55 |
|  |  |  | Richer | 55 | 55 | 55 |
|  |  |  | Richest | 55 | 55 | 55 |
|  |  |  | **Level of Education** |  |  |  |
|  | 1991 | 3871 | None | 39 | 39 | 39 |
|  |  |  | Primary | 39 | 39 | 39 |
|  |  |  | Secondary | 39 | 39 | 39 |
|  |  |  | Higher | 39 | 39 | 39 |
|  | 1998 | 5501 | None | 44 | 44 | 44 |
|  |  |  | Primary | 44 | 44 | 44 |
|  |  |  | Secondary | 44 | 44 | 44 |
|  |  |  | Higher | 44 | 44 | 44 |
|  | 2004 | 10656 | None | 42 | 42 | 42 |
|  |  |  | Primary | 42 | 42 | 42 |
|  |  |  | Secondary | 42 | 42 | 42 |
|  |  |  | Higher | 42 | 42 | 42 |
|  | 2011 | 15426 | None | 44 | 44 | 44 |
|  |  |  | Primary | 44 | 44 | 44 |
|  |  |  | Secondary | 44 | 44 | 44 |
|  |  |  | Higher | 44 | 44 | 44 |
|  | 2018 | 13500 | None | 44 | 44 | 44 |
|  |  |  | Primary | 44 | 44 | 44 |
|  |  |  | Secondary | 44 | 44 | 44 |
|  |  |  | Higher | 44 | 44 | 44 |

mCPR=modern contraceptive prevalence; mUNFP=unmet need for modern methods; mDSFP=demand satisfied with modern methods. Sample= women aged 15 to 49 years who are married or in a union
