# Supplementary material for: A subnational socioeconomic assessment of family planning levels, projections, and disparities among married women of reproductive age in Cameroon
Source: PLoS One. 2025 Feb 14;20(2):e0318650. doi: 10.1371/journal.pone.0318650 (PMC11828404; doi:10.1371/journal.pone.0318650)
Supplement: S2 Table — Married women include those who are married or in a union. Total number of women with a demand for family planning (modern contraceptive prevalence + traditional contraceptive prevalence + unmet need). Modern contraceptive methods include sterilisations, oral contraceptive pills, intrauterine devices, injectables, implants, condoms, lactational amenorrhea method, standard days method, emergency contraception and vaginal barrier methods. (DOCX) [file pone.0318650.s002.docx]

**S2 Table: Definitions of the coverage for family planning indicators**

| Family planning indicator | Calculations | |
| --- | --- | --- |
|  | **Numerator** | **Denominator** |
| Modern contraceptive prevalence | Currently married women aged 15–49 years using a modern method of contraception | Currently married women aged 15–49 years |
| Demand satisfied for family planning with modern methods | Currently married women aged 15–49 years with a demand for family planning, who are using a modern method of contraception | Currently married women aged 15–49 years, who have a demand for family planning |
| Unmet need for modern methods of family planning | Currently married women aged 15–49 years, who are not using a modern method of contraception and have a need for spacing or limiting | Currently married women aged 15–49 years |

Married women include those who are married or in a union. Total number of women with a demand for family planning (modern contraceptive prevalence + traditional contraceptive prevalence + unmet need). Modern contraceptive methods include sterilisations, oral contraceptive pills, intrauterine devices, injectables, implants, condoms, lactational amenorrhea method, standard days method, emergency contraception and vaginal barrier methods.
