# Supplementary material for: A subnational socioeconomic assessment of family planning levels, projections, and disparities among married women of reproductive age in Cameroon
Source: PLoS One. 2025 Feb 14;20(2):e0318650. doi: 10.1371/journal.pone.0318650 (PMC11828404; doi:10.1371/journal.pone.0318650)
Supplement: S3 Table — Estimates are in % (95% Credible Interval) (DOCX) [file pone.0318650.s003.docx]

**S3 Table: Use of, unmet need, and demand satisfied for modern contraceptive methods by rural and urban residence across regions of Cameroon, 2015 and 2030**

| **COUNTRY**  **Region** | **Urban(U)/**  **Rural(R)** | **Modern contraceptive prevalence** | | **Unmet need for modern methods** | | **Demand satisfied with modern methods** | |
| --- | --- | --- | --- | --- | --- | --- | --- |
|  |  | **2015** | **2030** | **2015** | **2030** | **2015** | **2030** |
| **CAMEROON** | U | 21.5 (9.2–43.8) | 36.9 (17.1–61.7) | 21.8 (13.8–32.4) | 21.7 (13.7–32.5) | 42.2 (22.5–65.0) | 62.6 (39.3–80.8) |
|  | R | 8.7 (3.2–21.0) | 16.4 (6.5–35.6) | 22.5 (14.4–33.7) | 22.4 (14.3–33.4) | 25.9 (12.1–47.0) | 44.0 (23.2–66.9) |
| **Adamawa** | U | 13.8 (5.4–31.5) | 27.4 (11.7–51.5) | 27.3 (17.7–39.2) | 25.6 (16.6–37.0) | 33.1 (16.2–56.1) | 55.6 (32.8–75.7) |
|  | R | 3.1 (1.1–8.3) | 6.9 (2.5–17.0) | 21.6 (13.6–32.3) | 20.1 (12.6–30.1) | 12.5 (5.4–26.9) | 26.4 (12.3–47.5) |
| **Centre** | U | 32.6 (14.7–58.1) | 60.7 (35.9–81.9) | 23.3 (15.1–34.3) | 21.8 (13.8–32.5) | 53.4 (30.9–74.7) | 77.9 (58.2–90.1) |
|  | R | 16.4 (6.7–35.9) | 38.3 (18.2–63.3) | 26.7 (17.3–38.4) | 25.0 (16.1–36.6) | 35.3 (17.7–58.3) | 62.5 (39.3–80.8) |
| **East** | U | 25.5 (10.9–49.4) | 47.5 (24.3–72.2) | 18.7 (11.6–28.5) | 13.4 (8.2–21.3) | 44.5 (24.0–67.1) | 67.9 (45.4–84.5) |
|  | R | 9.9 (3.7–23.6) | 22.5 (9.4–44.8) | 20.8 (13.1–31.3) | 15.1 (9.4–23.3) | 24.9 (11.6–46.0) | 46.6 (25.4–68.7) |
| **Far North** | U | 17.3 (6.8–37.4) | 49.2 (25.7–73.2) | 27.3 (17.8–39.4) | 28.5 (18.5–40.7) | 39.3 (20.3–62.6) | 73.5 (52.5–87.6) |
|  | R | 1.7 (0.6–4.9) | 7.6 (2.8–19.0) | 20.3 (12.7–30.3) | 21.0 (13.3–31.8) | 7.8 (3.2–17.7) | 26.7 (12.6–47.7) |
| **Littoral** | U | 19.1 (7.7–39.1) | 27.3 (11.7–51.3) | 21.1 (13.3–31.9) | 24.9 (15.9–36.7) | 35.8 (17.9–58.1) | 48.0 (26.4–70.1) |
|  | R | 13.3 (5.2–29.5) | 19.4 (7.9–40.5) | 29.8 (19.9–42.5) | 34.3 (22.9–47.5) | 28.0 (13.5–49.1) | 39.1 (20.2–62.0) |
| **Northwest** | U | 26.9 (11.4–51.0) | 49.7 (26.1–73.7) | 19.0 (12.0–28.9) | 18.2 (11.5–28.2) | 47.2 (25.9–70.0) | 71.0 (49.0–86.3) |
|  | R | 17.9 (7.5–38.1) | 36.9 (17.3–62.0) | 22.5 (14.2–33.4) | 21.6 (13.6–32.6) | 38.8 (20.3–61.2) | 63.3 (40.0–81.4) |
| **North** | U | 17.7 (7.0–38.3) | 56.6 (31.7–78.7) | 27.0 (17.5–39.1) | 29.5 (19.5–42.3) | 41.2 (21.7–64.8) | 81.6 (63.5–91.9) |
|  | R | 1.7 (0.6–4.8) | 9.5 (3.5–23.2) | 22.4 (14.2–33.5) | 24.7 (15.9–36.7) | 8.4 (3.5–18.8) | 36.3 (18.3–59.8) |
| **West** | U | 19.3 (7.7–39.8) | 30.3 (13.2–55.0) | 16.7 (10.2–26.2) | 15.4 (9.4–23.9) | 41.0 (21.4–63.6) | 60.3 (37.1–79.3) |
|  | R | 13.7 (5.4–30.8) | 22.3 (8.9–45.0) | 19.8 (12.5–29.9) | 18.3 (11.5–27.9) | 31.0 (15.4–53.3) | 49.3 (27.5–71.5) |
| **South** | U | 21.1 (8.6–43.4) | 29.8 (12.8–54.1) | 23.5 (15.1–34.5) | 24.5 (15.9–36.0) | 40.2 (20.9–63.3) | 52.0 (29.7–73.4) |
|  | R | 12.7 (4.9–29.3) | 18.7 (7.2–38.5) | 29.5 (19.5–42.3) | 30.8 (20.3–43.4) | 29.0 (13.7–51.2) | 39.8 (20.1–62.0) |
| **Southwest** | U | 22.1 (9.0–44.3) | 32.6 (14.7–57.9) | 16.9 (10.5–26.2) | 14.2 (8.7–22.1) | 41.5 (21.6–64.2) | 61.0 (37.8–79.9) |
|  | R | 16.5 (6.5–35.5) | 25.2 (10.7–48.6) | 18.9 (11.9–29.0) | 16.0 (9.9–24.8) | 37.8 (18.9–60.6) | 57.1 (34.2–77.0) |

Estimates are in % (95% Credible Interval)
