# Supplementary material for: A subnational socioeconomic assessment of family planning levels, projections, and disparities among married women of reproductive age in Cameroon
Source: PLoS One. 2025 Feb 14;20(2):e0318650. doi: 10.1371/journal.pone.0318650 (PMC11828404; doi:10.1371/journal.pone.0318650)
Supplement: S4 Table — Estimates are in % (95% Credible Interval); Q1 = poorest, Q2 = poorer, Q3 = middle, Q4 = richer, Q5 = richest. (DOCX) [file pone.0318650.s004.docx]

**S4 Table: Use of, unmet need, and demand satisfied for modern contraceptive methods by wealth quintile across regions of Cameroon, 2015 and 2030**

| **COUNTRY**  **Region** | **Wealth Quintile** | **Modern contraceptive prevalence** | | **Unmet need for modern methods** | | **Demand satisfied with modern methods** | |
| --- | --- | --- | --- | --- | --- | --- | --- |
|  |  | **2015** | **2030** | **2015** | **2030** | **2015** | **2030** |
| **CAMEROON** | Q1 | 2.7 (0.7–9.8) | 5.3 (1.4–17.6) | 10.3 (3.9–25.5) | 6.4 (2.3–16.4) | 14.0 (4.4– 36.4) | 27.5 (9.7–56.2) |
|  | Q2 | 5.0 (1.3–16.8) | 9.5 (2.6–28.6) | 11.2 (4.3–27.2) | 7.1 (2.5–18.2) | 18.8 (6.1– 44.2) | 34.9 (13.5–65.1) |
|  | Q3 | 9.6 (2.7–29.4) | 17.3 (5.1–45.0) | 12.4 (4.7–29.4) | 7.8 (2.7–20.0) | 26.8 (9.8– 56.7) | 45.8 (19.3–74.8) |
|  | Q4 | 13.6 (4.0–37.6) | 23.9 (7.3–53.6) | 10.9 (4.1–26.5) | 6.9 (2.5–17.5) | 32.6 (12.4– 62.6) | 53.1 (24.1–79.1) |
|  | Q5 | 19.8 (5.9–48.6) | 33.0 (11.2–65.9) | 9.4 (3.4–23.1) | 5.8 (2.0–15.1) | 40.5 (16.0– 70.4) | 61.4 (31.8–84.8) |
| **Adamawa** | Q1 | 6.2 (1.7–20.5) | 18.4 (5.5–46.5) | 11.0 (4.1–26.5) | 6.8 (2.4–17.6) | 28.6 (10.5– 58.6) | 68.3 (38.0–88.2) |
|  | Q2 | 4.4 (1.2–15.1) | 13.7 (4.0–38.8) | 8.4 (3.1–20.7) | 5.0 (1.8–13.1) | 27.8 (10.0– 56.8) | 67.4 (36.9–88.2) |
|  | Q3 | 3.9 (1.0–13.4) | 12.1 (3.4–33.9) | 7.9 (2.9–19.9) | 4.8 (1.7–12.7) | 21.2 (7.2– 48.2) | 58.9 (29.3–83.0) |
|  | Q4 | 9.8 (2.7–30.0) | 27.1 (9.0–58.2) | 10.6 (3.9–25.5) | 6.5 (2.3–16.5) | 37.3 (14.4– 68.1) | 76.0 (48.5–91.6) |
|  | Q5 | 32.7 (10.9–65.5) | 62.6 (30.0–86.4) | 13.3 (5.2–30.8) | 8.3 (3.0–21.0) | 65.6 (34.3– 87.2) | 91.2 (74.6–97.3) |
| **Centre** | Q1 | 9.9 (2.8–30.3) | 24.2 (7.7–55.4) | 8.8 (3.3–21.9) | 8.1 (3.0–20.7) | 34.6 (13.6– 65.0) | 59.0 (29.4–83.5) |
|  | Q2 | 7.8 (2.1–24.1) | 19.6 (5.9–48.5) | 15.1 (5.6–34.3) | 13.8 (5.2–31.7) | 20.4 (6.8– 46.7) | 40.9 (16.6– 70.6) |
|  | Q3 | 9.3 (2.6–27.8) | 22.6 (7.0–52.8) | 12.3 (4.7–28.7) | 11.2 (4.2–26.9) | 24.4 (8.6– 52.4) | 46.6 (20.1– 75.0) |
|  | Q4 | 15.2 (4.5–40.9) | 34.4 (11.9–66.9) | 11.0 (4.1–25.8) | 10.0 (3.8–24.1) | 34.0 (13.0– 64.2) | 58.5 (29.2– 83.1) |
|  | Q5 | 20.5 (6.3–50.4) | 43.0 (16.3–73.8) | 9.8 (3.7–24.1) | 9.1 (3.3–21.9) | 40.5 (16.3– 70.4) | 65.1 (34.9–86.3) |
| **East** | Q1 | 1.6 (0.4–5.8) | 1.7 (0.4–6.2) | 8.2 (2.9–21.1) | 4.5 (1.6–11.8) | 5.2 (1.6– 15.9) | 4.9 (1.4–15.2) |
|  | Q2 | 4.5 (1.2–15.0) | 4.6 (1.2–15.5) | 12.6 (4.7–29.0) | 6.9 (2.5–17.4) | 12.9 (4.1– 33.4) | 12.0 (3.7–31.7) |
|  | Q3 | 5.5 (1.5–18.6) | 5.9 (1.6–19.0) | 10.6 (4.0–25.1) | 5.9 (2.1–15.0) | 13.8 (4.3– 35.7) | 13.0 (4.1–33.6) |
|  | Q4 | 5.7 (1.5–19.4) | 6.0 (1.6–19.6) | 9.8 (3.6–24.1) | 5.4 (1.9–14.5) | 14.2 (4.6– 37.0) | 13.3 (4.3–34.9) |
|  | Q5 | 10.0 (2.8–29.6) | 10.4 (3.0–31.2) | 7.2 (2.6–18.5) | 3.9 (1.4–10.6) | 21.2 (7.3– 48.0) | 19.8 (6.8–46.7) |
| **Far North** | Q1 | 2.0 (0.5–7.2) | 4.2 (1.1–14.2) | 8.8 (3.2–21.8) | 4.7 (1.6–12.7) | 12.2 (3.9– 32.6) | 24.3 (8.4–52.4) |
|  | Q2 | 2.5 (0.7–9.3) | 5.3 (1.5–17.3) | 10.1 (3.7–24.5) | 5.4 (1.9–14.2) | 12.2 (3.8– 33.0) | 24.2 (8.6–52.2) |
|  | Q3 | 4.1 (1.1–14.0) | 8.2 (2.4–26.2) | 12.2 (4.4–28.9) | 6.6 (2.3–16.9) | 15.5 (4.9– 38.5) | 29.3 (11.1–59.7) |
|  | Q4 | 6.0 (1.6–19.7) | 11.9 (3.4–34.3) | 9.9 (3.6–23.6) | 5.2 (1.8–14.0) | 19.8 (6.6– 46.2) | 36.2 (13.9–66.5) |
|  | Q5 | 16.4 (4.7–41.8) | 29.1 (9.6–61.8) | 10.5 (3.8–25.3) | 5.6 (2.0–14.7) | 41.7 (16.6– 70.1) | 61.8 (32.0–85.1) |
| **Littoral** | Q1 | 7.3 (2.1–23.1) | 9.1 (2.6–27.5) | 23.3 (9.5–46.5) | 20.7 (8.1–43.5) | 16.8 (5.7– 41.1) | 22.8 (8.0–50.3) |
|  | Q2 | 6.6 (1.8–21.6) | 8.2 (2.3–26.2) | 17.2 (6.5–37.4) | 15.2 (5.9–33.8) | 18.7 (6.3– 44.7) | 25.2 (8.8–54.7) |
|  | Q3 | 11.4 (3.2–33.7) | 13.8 (4.0–37.8) | 18.7 (7.3–40.1) | 16.6 (6.4–36.9) | 26.6 (9.4– 56.6) | 34.3 (13.2–64.3) |
|  | Q4 | 8.8 (2.4–27.1) | 10.7 (3.0–31.8) | 10.8 (4.1–26.3) | 9.5 (3.5–23.2) | 23.7 (8.2– 52.6) | 31.1 (11.4–61.0) |
|  | Q5 | 13.1 (3.9–36.5) | 16.1 (4.8–43.1) | 9.3 (3.3–22.6) | 7.9 (3.0–20.4) | 31.6 (12.0– 61.7) | 40.5 (16.5–70.9) |
| **Northwest** | Q1 | 7.0 (1.9–22.6) | 10.5 (3.0–31.4) | 11.5 (4.3–27.2) | 6.5 (2.3–16.5) | 24.6 (8.6– 54.0) | 36.9 (14.3–67.1) |
|  | Q2 | 5.7 (1.6–19.2) | 8.6 (2.3–27.1) | 11.0 (3.9–25.8) | 6.1 (2.2–16.2) | 18.8 (6.4– 45.0) | 29.2 (10.4–59.4) |
|  | Q3 | 12.5 (3.6–35.3) | 18.2 (5.3–46.3) | 10.7 (3.9–26.1) | 6.0 (2.2–15.8) | 28.1 (10.0– 57.4) | 41.0 (16.5–70.4) |
|  | Q4 | 17.4 (5.2–45.4) | 24.8 (7.9–56.4) | 9.8 (3.5–23.8) | 5.4 (1.9–14.0) | 36.9 (14.5– 67.4) | 51.1 (23.3–78.1) |
|  | Q5 | 22.1 (6.7–51.6) | 30.7 (10.6–63.4) | 8.3 (3.0–20.0) | 4.6 (1.6–12.5) | 39.9 (15.7– 69.1) | 54.2 (25.8–80.6) |
| **North** | Q1 | 1.4 (0.4–5.0) | 4.1 (1.1–14.5) | 10.5 (3.9–25.8) | 7.6 (2.8–19.2) | 8.4 (2.6– 23.8) | 25.2 (8.9–54.5) |
|  | Q2 | 2.3 (0.6–8.4) | 6.8 (1.9–21.6) | 7.9 (2.8–19.4) | 5.7 (2.0–14.9) | 13.8 (4.3– 35.7) | 37.0 (14.7–66.9) |
|  | Q3 | 3.0 (0.8–10.9) | 8.8 (2.5–26.9) | 10.7 (4.0–25.8) | 7.7 (2.8–19.3) | 15.5 (5.1– 39.5) | 40.5 (16.4–69.8) |
|  | Q4 | 7.4 (2.0–23.7) | 20.2 (6.0–49.3) | 13.8 (5.2–31.1) | 10.0 (3.7–23.9) | 24.7 (8.6– 52.9) | 55.3 (25.9–81.4) |
|  | Q5 | 18.6 (5.6–46.9) | 41.4 (15.4–73.2) | 13.9 (5.3–31.6) | 10.1 (3.8–24.0) | 40.7 (16.8– 70.5) | 71.8 (42.3–89.9) |
| **West** | Q1 | 7.2 (2.0–22.8) | 10.0 (2.8–29.7) | 12.0 (4.5–27.9) | 6.8 (2.5–17.7) | 20.1 (6.7– 46.4) | 31.6 (11.8–61.3) |
|  | Q2 | 7.0 (1.9–22.8) | 9.7 (2.8–28.8) | 9.9 (3.7–24.1) | 5.6 (2.0–14.6) | 20.0 (6.6– 46.9) | 31.4 (11.7–61.2) |
|  | Q3 | 9.4 (2.6–28.2) | 13.1 (3.7–36.4) | 12.3 (4.8–28.7) | 7.1 (2.6–18.0) | 25.0 (8.9– 53.5) | 38.2 (14.9–67.7) |
|  | Q4 | 14.8 (4.3–39.8) | 20.2 (5.9–49.3) | 9.4 (3.4–22.8) | 5.3 (1.9–14.0) | 34.4 (13.2– 64.1) | 49.4 (21.9–76.9) |
|  | Q5 | 17.5 (5.3–45.6) | 23.6 (7.4–53.4) | 6.5 (2.3–16.6) | 3.7 (1.3–10.0) | 37.0 (14.7– 67.4) | 52.2 (23.8–78.6) |
| **South** | Q1 | 7.1 (2.0–22.7) | 9.5 (2.7–28.8) | 12.9 (4.8–30.2) | 8.1 (2.9–19.9) | 25.6 (8.9– 54.8) | 39.0 (15.6–68.7) |
|  | Q2 | 8.6 (2.3–26.5) | 11.3 (3.1–32.7) | 9.3 (3.3–23.4) | 5.7 (2.1–15.2) | 30.1 (10.8– 60.1) | 44.2 (18.4–73.6) |
|  | Q3 | 11.1 (3.0–32.0) | 14.6 (4.2–40.0) | 12.5 (4.8–29.7) | 7.9 (2.8–19.9) | 31.2 (11.2– 60.9) | 45.8 (19.1–74.6) |
|  | Q4 | 10.0 (2.8–29.6) | 13.3 (3.9–36.5) | 10.0 (3.7–24.3) | 6.2 (2.2–16.3) | 31.3 (11.6– 61.3) | 46.1 (19.6–74.3) |
|  | Q5 | 14.3 (4.2–39.6) | 18.6 (5.7–47.2) | 7.7 (2.8–19.3) | 4.7 (1.6–12.6) | 36.4 (14.1– 67.0) | 51.7 (23.8–79.4) |
| **Southwest** | Q1 | 13.4 (3.7–37.9) | 16.8 (5.1–43.1) | 10.0 (3.6–24.5) | 5.9 (2.1–15.4) | 33.6 (12.1– 64.2) | 46.1 (20.0–74.0) |
|  | Q2 | 6.7 (1.8–22.1) | 8.7 (2.4–27.1) | 11.1 (4.0–26.4) | 6.6 (2.4–17.2) | 19.0 (6.3– 45.2) | 28.7 (10.1–58.8) |
|  | Q3 | 11.7 (3.3–35.0) | 15.0 (4.3–40.5) | 14.4 (5.5–33.6) | 8.7 (3.1–21.8) | 27.8 (9.9– 57.9) | 39.8 (15.9–69.8) |
|  | Q4 | 13.7 (3.8–37.3) | 17.3 (5.3–44.5) | 9.9 (3.6–23.9) | 5.8 (2.1–15.2) | 29.5 (10.3– 58.8) | 41.7 (17.2–71.3) |
|  | Q5 | 18.4 (5.6–47.0) | 23.4 (7.3–53.6) | 10.0 (3.6–24.5) | 5.9 (2.1–15.3) | 37.8 (14.8– 68.2) | 51.4 (22.8–78.4) |

Estimates are in % (95% Credible Interval); Q1=poorest, Q2=poorer, Q3=middle, Q4=richer, Q5=richest.
