# Supplementary material for: A subnational socioeconomic assessment of family planning levels, projections, and disparities among married women of reproductive age in Cameroon
Source: PLoS One. 2025 Feb 14;20(2):e0318650. doi: 10.1371/journal.pone.0318650 (PMC11828404; doi:10.1371/journal.pone.0318650)
Supplement: S5 Table — %p = percentage points. (DOCX) [file pone.0318650.s005.docx]

**S5 Table: Magnitude of socio-economic inequalities in the use of, unmet need, and demand satisfied for modern contraceptive methods across regions of Cameroon, 2015 and 2030**

| **COUNTRY**  **Region** | **Slope Index of Inequality (%p)** | | | |
| --- | --- | --- | --- | --- |
|  | **Wealth-based** | | **Education-based** | |
|  | Modern contraceptive use | | Modern contraceptive use | |
|  | **2015** | **2030** | **2015** | **2030** |
| CAMEROON | 22.5 (20.3–24.7) | 35.5 (32.3–38.7) | 47.0 (35.4–58.7) | 60.1 (46.6–73.7) |
| Adamawa | 31.4 (10.7–52.0) | 49.7 (16.4–83.0) | 64.9 (62.2–67.7) | 75.8 (72.5–79.2) |
| Centre | 14.4 (7.3–21.6) | 26.0 (12.2–39.8) | 39.7 (37.6–41.8) | 54.7 (52.5–56.9) |
| East | 9.4 (6.4–12.4) | 9.8 (6.8–12.8) | 32.2 (20.7–43.8) | 50.6 (36.8–64.3) |
| Far North | 18.1 (11.9–24.3) | 30.0 (20.2–39.9) | 50.0 (34.0–66.0) | 64.3 (46.8–81.7) |
| Littoral | 6.9 (3.6–10.2) | 8.3 (4.2–12.4) | 25.3 (17.6–33.1) | 28.7 (20.0–37.5) |
| Northwest | 21.5 (16.8–26.1) | 28.4 (22.1–34.8) | 29.1 (25.5–32.8) | 39.6 (35.2–44.1) |
| North | 23.1 (18.3–27.9) | 46.0 (38.0–54.1) | 57.0 (44.4–69.5) | 72.2 (59.1–85.2) |
| West | 14.5 (11.4–17.5) | 18.9 (14.8–23.1) | 39.0 (35.2–42.8) | 46.5 (42.7–50.3) |
| South | 7.9 (5.4–10.4) | 10.2 (7.2–13.2) | 25.1 (15.3–34.9) | 33.0 (20.5–45.4) |
| Southwest | 8.5 (-2.2–19.3) | 10.9 (-2.3–24.1) | 7.5 (-6.0–21.1) | 9.6 (-8.9–28.0) |
|  | Unmet need for modern methods | | Unmet need for modern methods | |
|  | **2015** | **2030** | **2015** | **2030** |
| CAMEROON | -1.2 (-4.3–1.9) | -0.7 (-3.0–1.5) | -17.0 (-33.6– -0.4) | -18.6 (-36.1– -1.1) |
| Adamawa | 3.4 (-2.6–9.5) | 2.2 (-1.6–6.1) | 30.7 (3.2–58.2) | 30.6 (3.2–57.9) |
| Centre | -1.0 (-9.1–7.0) | -0.9 (-8.1–6.4) | -11.3 (-30.9–8.2) | -11.5 (-31.4–8.4) |
| East | -2.3 (-9.2–4.6) | -1.3 (-5.2–2.5) | -3.3 (-16.0–9.3) | -2.9 (-13.0–7.3) |
| Far North | 1.6 (-0.8–3.9) | 0.8 (-0.5–2.0) | -7.7 (-18.2–2.9) | -8.8 (-20.5–3.0) |
| Littoral | -17.3 (-19.8– -14.7) | -15.7 (-18.1– -13.3) | -31.1 (-38.9– -23.3) | -35.3 (-44.2– -26.4) |
| Northwest | -3.8 (-5.2– -2.5) | -2.2 (-2.9– -1.5) | -7.3 (-14.7–0.1) | -7.4 (-15.2–0.4) |
| North | 6.3 (1.9–10.7) | 4.6 (1.4–7.9) | -26.8 (-50.8– -2.7) | -30.8 (-58.0– -3.7) |
| West | -5.7 (-9.2– -2.2) | -3.3 (-5.3– -1.2) | -0.7 (-11.4–10.0) | -0.7 (-10.9–9.5) |
| South | -4.9 (-8.2– -1.6) | -3.2 (-5.3– -1.0) | 4.8 (-18.3–27.9) | 5.2 (-19.7–30.1) |
| Southwest | -0.6 (-4.3–3.0) | -0.4 (-2.8–1.9) | 9.3 (1.4–17.3) | 8.1 (0.9–15.3) |

%p= percentage points.
