# Supplementary material for: A subnational socioeconomic assessment of family planning levels, projections, and disparities among married women of reproductive age in Cameroon
Source: PLoS One. 2025 Feb 14;20(2):e0318650. doi: 10.1371/journal.pone.0318650 (PMC11828404; doi:10.1371/journal.pone.0318650)
Supplement: S6 Table — Estimates are in % (95% Credible Interval); E1 = none, E2 = primary, E3 = secondary, E4 = higher levels of educational attainment. (DOCX) [file pone.0318650.s006.docx]

**S6 Table: Use of, unmet need, and demand satisfied for modern contraceptive methods by level of education across regions of Cameroon, 2015 and 2030**

| **COUNTRY**  **Region** | **Level of Education** | **Modern contraceptive prevalence** | | **Unmet need for modern methods** | | **Demand satisfied with modern methods** | |
| --- | --- | --- | --- | --- | --- | --- | --- |
|  |  | **2015** | **2030** | **2015** | **2030** | **2015** | **2030** |
| **CAMEROON** | E1 | 3.1 (1.2–7.9) | 5.5 (2.1–13.6) | 27.0 (14.8–44.9) | 30.3 (16.6–49.1) | 13.2 (5.5–29.5) | 24.1 (10.5–46.6) |
|  | E2 | 14.3 (5.9–30.9) | 23.0 (10.3–43.9) | 30.7 (16.9–49.1) | 34.0 (19.4–52.5) | 33.0 (15.5–57.0) | 50.3 (27.7–72.8) |
|  | E3 | 27.0 (12.1–50.1) | 39.9 (19.7–64.2) | 26.1 (13.9–43.8) | 29.2 (15.8–47.5) | 45.9 (23.8–69.9) | 63.5 (38.9–82.6) |
|  | E4 | 37.9 (19.0–61.7) | 52.5 (29.0–74.7) | 14.3 (7.2–26.5) | 16.4 (8.2–30.0) | 58.1 (34.5–78.7) | 74.2 (51.3–88.6) |
| **Adamawa** | E1 | 2.3 (0.9–6.0) | 4.3 (1.7–10.9) | 27.5 (14.9–45.1) | 28.0 (15.4–46.0) | 9.6 (3.8–22.0) | 19.6 (8.5–39.9) |
|  | E2 | 10.7 (4.3–24.3) | 18.7 (7.9–38.0) | 28.5 (15.5–46.5) | 29.2 (15.9–47.3) | 30.5 (14.0–54.3) | 50.7 (27.5–73.4) |
|  | E3 | 24.5 (10.8–46.2) | 38.0 (18.5–63.0) | 25.2 (13.4–42.2) | 25.7 (13.6–43.7) | 47.5 (25.1–70.7) | 67.7 (43.4–85.4) |
|  | E4 | 52.4 (29.2–75.0) | 67.4 (44.0–84.7) | 54.5 (35.5–72.5) | 55.0 (36.2–72.7) | 78.7 (57.9–91.0) | 89.5 (76.1–95.8) |
| **Centre** | E1 | 9.3 (3.7–21.8) | 25.3 (11.5–47.4) | 22.6 (11.7–39.2) | 23.2 (12.4–39.6) | 29.4 (13.3–53.4) | 55.9 (32.5–77.3) |
|  | E2 | 16.0 (6.7–33.3) | 38.7 (19.0–63.0) | 30.1 (16.5–48.0) | 30.9 (16.9–49.5) | 31.9 (14.8–55.3) | 58.8 (34.4–79.5) |
|  | E3 | 27.3 (12.3–50.7) | 55.5 (32.1–76.9) | 25.8 (13.8–43.6) | 26.5 (14.4–44.0) | 44.4 (22.9–68.9) | 70.9 (47.8–86.8) |
|  | E4 | 38.8 (19.3–62.6) | 67.8 (44.1–84.9) | 14.5 (7.3–26.9) | 15.0 (7.5–27.8) | 58.6 (34.6–79.1) | 81.3 (61.7–92.1) |
| **East** | E1 | 5.4 (2.1–13.4) | 13.0 (5.2–28.4) | 16.6 (8.3–30.4) | 12.6 (6.1–23.8) | 19.0 (7.9–39.0) | 32.8 (15.0–56.6) |
|  | E2 | 9.1 (3.6–20.8) | 20.8 (9.1–41.3) | 23.1 (12.0–39.3) | 18.0 (9.2–32.3) | 19.0 (7.9–38.4) | 33.0 (15.7–57.1) |
|  | E3 | 24.9 (11.0–46.4) | 45.9 (24.4–69.7) | 20.4 (10.5–35.5) | 15.5 (7.8–28.7) | 37.4 (18.0–61.2) | 54.9 (31.3–76.8) |
|  | E4 | 26.4 (11.8–48.9) | 48.3 (25.9–71.3) | 14.7 (7.3–27.2) | 11.0 (5.4–21.2) | 46.5 (24.3–70.0) | 64.3 (40.0–82.8) |
| **Far North** | E1 | 1.0 (0.4–2.6) | 1.7 (0.6–4.4) | 25.6 (13.4–42.9) | 31.1 (17.1–49.6) | 5.5 (2.1–13.6) | 11.8 (4.7–26.5) |
|  | E2 | 7.7 (3.1–18.2) | 12.8 (5.2–28.3) | 25.0 (13.3–41.9) | 30.6 (16.9–49.2) | 30.2 (13.9–53.6) | 50.2 (27.2–73.2) |
|  | E3 | 27.2 (12.3–49.6) | 39.3 (19.1–63.1) | 28.0 (15.2–45.6) | 33.7 (18.6–52.4) | 49.1 (26.5–72.0) | 68.9 (44.5–85.5) |
|  | E4 | 34.7 (16.9–58.8) | 48.3 (25.9–71.6) | 18.2 (9.4–32.7) | 22.8 (11.9–39.3) | 27.4 (12.5–50.6) | 46.9 (24.7–70.6) |
| **Littoral** | E1 | 9.7 (3.8–22.2) | 11.9 (4.8–27.0) | 35.1 (19.7–54.0) | 41.5 (24.4–61.1) | 26.1 (11.4–48.6) | 34.3 (16.1–59.0) |
|  | E2 | 10.1 (4.0–22.9) | 12.5 (5.2–28.0) | 28.9 (15.7–46.8) | 35.0 (20.0–54.2) | 24.3 (10.6–46.1) | 32.3 (15.3–56.7) |
|  | E3 | 16.6 (6.7–34.8) | 20.1 (8.6–40.6) | 21.8 (11.1–37.8) | 26.7 (14.4–44.7) | 33.2 (15.2–57.4) | 42.3 (21.4–66.9) |
|  | E4 | 28.3 (12.9–51.2) | 33.3 (15.7–56.9) | 11.4 (5.6–21.8) | 14.4 (7.2–26.6) | 49.8 (26.9–72.7) | 59.4 (35.1–79.6) |
| **Northwest** | E1 | 7.1 (2.7–17.0) | 11.9 (4.8–26.7) | 22.3 (11.5–38.5) | 23.6 (12.4–40.3) | 22.2 (9.4–43.5) | 37.4 (18.2–62.0) |
|  | E2 | 11.4 (4.6–25.4) | 18.6 (7.9–38.1) | 21.0 (10.8–36.5) | 22.3 (11.6–38.6) | 28.1 (12.6–51.1) | 45.3 (23.4–69.2) |
|  | E3 | 21.0 (9.2–42.2) | 32.2 (15.4–55.7) | 13.9 (7.0–26.5) | 14.9 (7.6–27.5) | 37.1 (18.2–62.1) | 55.6 (32.2–76.9) |
|  | E4 | 27.8 (12.6–50.9) | 40.8 (20.6–65.0) | 18.5 (9.4–33.3) | 19.8 (10.2–35.1) | 39.8 (19.6–64.3) | 58.5 (34.4–79.2) |
| **North** | E1 | 1.8 (0.7–4.5) | 3.3 (1.3–8.2) | 26.7 (14.1–44.0) | 31.7 (17.6–50.0) | 8.6 (3.3–20.1) | 22.1 (9.6–43.0) |
|  | E2 | 4.7 (1.8–11.5) | 8.4 (3.4–19.6) | 28.7 (15.9–46.7) | 33.9 (19.2–52.6) | 18.2 (7.8–37.4) | 40.1 (20.1–64.2) |
|  | E3 | 27.9 (12.5–51.7) | 42.5 (21.9–66.7) | 24.3 (12.7–41.7) | 29.2 (16.0–47.6) | 55.5 (31.4–77.7) | 79.3 (58.9–91.3) |
|  | E4 | 39.9 (20.1–63.8) | 55.8 (32.2–77.1) | 5.8 (2.8–11.8) | 7.3 (3.5–14.7) | 48.1 (25.8–71.3) | 73.9 (51.3–88.4) |
| **West** | E1 | 4.7 (1.8–11.7) | 6.5 (2.5–15.8) | 13.7 (6.9–25.7) | 13.0 (6.3–24.5) | 20.8 (9.0–41.5) | 33.4 (15.4–57.5) |
|  | E2 | 9.5 (3.9–22.0) | 13.0 (5.3–28.4) | 22.4 (12.0–38.6) | 21.4 (11.0–36.9) | 25.5 (11.6–48.0) | 39.5 (19.4–63.5) |
|  | E3 | 21.3 (9.2–42.5) | 27.4 (12.8–50.4) | 15.3 (7.7–28.6) | 14.4 (7.3–26.9) | 41.8 (21.1–66.4) | 57.4 (34.1–78.6) |
|  | E4 | 32.5 (15.2–56.3) | 40.6 (20.2–64.5) | 15.4 (7.7–28.5) | 14.7 (7.3–27.2) | 53.7 (29.9–75.8) | 68.9 (44.8–85.6) |
| **South** | E1 | 2.1 (0.8–5.3) | 3.1 (1.2–8.0) | 15.9 (7.9–28.8) | 18.6 (9.5–33.2) | 10.2 (4.0–23.1) | 13.5 (5.5–29.6) |
|  | E2 | 10.8 (4.3–24.1) | 15.3 (6.3–32.9) | 30.1 (16.4–48.1) | 33.9 (19.0–53.1) | 24.5 (10.7–46.2) | 30.4 (13.8–54.4) |
|  | E3 | 14.8 (6.0–31.8) | 20.6 (8.9–40.8) | 28.7 (15.5–46.9) | 32.5 (18.2–51.1) | 27.2 (12.1–50.4) | 33.5 (15.8–57.5) |
|  | E4 | 20.9 (9.0–41.1) | 28.3 (13.0–51.6) | 20.4 (10.5–35.5) | 23.4 (12.4–40.1) | 39.3 (19.4–63.3) | 46.6 (24.6–70.4) |
| **Southwest** | E1 | 21.3 (9.2–42.2) | 35.7 (17.0–60.1) | 10.8 (5.3–21.0) | 9.3 (4.4–18.5) | 62.4 (38.1–81.8) | 83.1 (64.2–93.1) |
|  | E2 | 15.9 (6.7–34.2) | 28.1 (12.8–51.1) | 19.9 (10.3–35.5) | 17.4 (8.8–31.5) | 36.4 (17.7–61.3) | 63.1 (38.9–82.2) |
|  | E3 | 18.5 (7.8–37.6) | 31.7 (14.8–55.7) | 14.6 (7.3–27.1) | 12.6 (6.2–24.2) | 36.7 (17.6–60.8) | 63.2 (38.8–82.4) |
|  | E4 | 26.7 (12.1–48.9) | 42.5 (21.3–66.3) | 20.3 (10.5–35.4) | 17.6 (8.8–31.7) | 53.9 (30.3–75.6) | 77.4 (55.4–90.2) |

Estimates are in % (95% Credible Interval); E1=none, E2=primary, E3=secondary, E4=higher levels of educational attainment.
