# Supplementary material for: A subnational socioeconomic assessment of family planning levels, projections, and disparities among married women of reproductive age in Cameroon
Source: PLoS One. 2025 Feb 14;20(2):e0318650. doi: 10.1371/journal.pone.0318650 (PMC11828404; doi:10.1371/journal.pone.0318650)
Supplement: S7 Table — Modern methods=Modern contraceptive methods; Media exposure represents a combination of variables for frequency of reading newspapers or magazines, listening to radio, watching television, and use of internet; CrI = Credible Interval. (DOCX) [file pone.0318650.s007.docx]

**S7 Table: Determinants of use of and unmet need for modern methods of famil****y planning in Cameroon, DHS 2018**

| **Variable** | **Categories** | **Odds Ratio (95% CrI)** | | | | **N (%)** |
| --- | --- | --- | --- | --- | --- | --- |
|  |  | **Crude** | | | **Adjusted** |  |
|  |  | Modern contraceptive use | Unmet need for modern methods | Demand satisfied with modern methods | Demand satisfied with modern methods |  |
| HIV status | Negative | 1 | 1 | 1 | 1 | 1,450 (95.8) |
|  | Positive | 1.05 (0.54, 1.80) | 0.98 (0.54, 1.79) | 1.10 (0.53, 2.03) | 0.77 (0.59, 1.00) | 63 (4.2) |
| Age group, years | 15–19 | 1 | 1 | 1 | 1 | 117 (7.7) |
|  | 20–29 | 2.69 (1.67, 4.74) | 0.30 (0.17, 0.51) | 3.81 (2.12, 6.98) | 2.66 (2.33, 3.00) | 584 (38.6) |
|  | 30–39 | 2.72 (1.69, 4.62) | 0.31 (0.16, 0.53) | 3.65 (2.01, 6.64) | 2.64 (2.11, 3.26) | 573 (37.9) |
|  | 40–49 | 2.04 (1.18, 3.65) | 0.40 (0.22, 0.76) | 2.70 (1.43, 4.88) | 1.96 (1.54, 2.51) | 239 (15.8) |
| Level of education | None | 1 | 1 | 1 | 1 | 253 (16.7) |
|  | Primary | 4.21 (2.61, 7.10) | 0.26 (0.17, 0.38) | 5.74 (3.32, 10.84) | 2.31 (1.78, 2.91) | 493 (32.6) |
|  | Secondary | 5.82 (3.77, 9.41) | 0.17 (0.11, 0.26) | 9.13 (5.48, 18.57) | 3.14 (2.71, 3.67) | 662 (43.8) |
|  | Higher | 5.48 (3.00, 10.65) | 0.13 (0.07, 0.23) | 13.36 (6.51, 33.31) | 2.78 (2.08, 3.62) | 105 (6.9) |
| Religion | Muslim | 1 | 1 | 1 | 1 | 317 (21.0) |
|  | Catholic | 2.04 (1.47, 2.86) | 0.42 (0.29, 0.59) | 2.72 (1.80, 4.34) | 1.43 (1.21, 1.66) | 581 (38.4) |
|  | Christian | 1.96 (1.42, 2.75) | 0.42 (0.29, 0.60) | 2.71 (1.73, 4.32) | 1.65 (1.47, 1.86) | 569 (37.6) |
|  | None/other | 0.42 (0.16, 1.06) | 1.31 (0.58, 3.07) | 0.76 (0.28, 1.92) | 0.53 (0.39, 0.69) | 46 (3.0) |
| Living children | None | 1 | 1 | 1 | 1 | 37 (2.4) |
|  | 1–3 | 1.98 (0.87, 4.98) | 0.66 (0.21, 1.88) | 1.45 (0.61, 3.54) | 1.17 (0.91, 1.46) | 762 (50.4) |
|  | 4–6 | 2.32 (1.02, 5.70) | 0.56 (0.18, 1.60) | 1.64 (0.69, 3.98) | 1.40 (1.07, 1.78) | 547 (36.2) |
|  | ≥6 | 1.29 (0.54, 3.40) | 1.45 (0.42, 4.96) | 0.73 (0.28, 1.91) | 0.86 (0.63, 1.19) | 167 (11.0) |
| Media exposure | None | 1 | 1.00 | 1.00 | 1 | 469 (31.0) |
|  | <Once a week | 2.00 (1.53, 2.62) | 0.38 (0.27, 0.52) | 2.60 (1.87, 3.65) | 1.18 (1.01, 1.38) | 602 (39.8) |
|  | >Once a week | 1.89 (1.40, 2.52) | 0.44 (0.31, 0.62) | 2.25 (1.57, 3.19) | 1.05 (0.80, 1.32) | 440 (29.1) |
| Wealth quintile | Poorest | 1 | 1 | 1 | 1 | 192 (12.7) |
|  | Poorer | 2.91 (1.77, 5.31 | 0.37 (0.23, 0.60) | 2.61 (1.56, 4.37) | 1.85 (1.44, 2.27) | 308 (20.4) |
|  | Middle | 3.85 (2.32, 7.19 | 0.29 (0.18, 0.47) | 3.33 (2.05, 5.43) | 2.18 (1.72, 2.66) | 383 (25.3) |
|  | Richer | 3.90 (2.36, 7.44 | 0.23 (0.14, 0.39) | 4.09 (2.38, 6.75) | 2.06 (1.68, 2.50) | 309 (20.4) |
|  | Richest | 5.70 (3.32, 11.24 | 0.15 (0.09, 0.24) | 6.37 (3.95, 10.53) | 3.24 (2.81, 3.75) | 321 (21.2) |
| Residence | Rural | 1 | 1 | 1 | 1 | 727 (48.1) |
|  | Urban | 1.61 (1.26, 2.03) | 0.49 (0.34, 0.67) | 1.97 (1.45, 2.70) | 1.09 (0.91, 1.30) | 786 (51.9) |
| Region | Adamawa | 1 | 1 | 1 | 1 | 95 (6.3) |
|  | Centre | 3.38 (1.64, 6.44) | 0.24 (0.12, 0.44) | 3.83 (1.97, 7.52) | 1.42 (1.18, 1.66) | 318 (21.0) |
|  | East | 4.16 (1.94, 8.26) | 0.08 (0.03, 0.16) | 10.87 (5.23, 22.59) | 7.81 (5.91, 10.10) | 166 (11.0) |
|  | Far North | 1.63 (0.75, 3.26) | 0.65 (0.33, 1.40) | 1.40 (0.81, 2.52) | 1.81 (1.45, 2.19) | 132 (8.7) |
|  | Littoral | 2.97 (1.56, 5.68) | 0.25 (0.12, 0.46) | 3.78 (1.99, 6.50) | 1.01 (0.78, 1.26) | 213 (14.1) |
|  | Northwest | 1.63 (0.85, 3.13) | 0.67 (0.32, 1.36) | 1.29 (0.56, 2.52) | 1.46 (1.18, 1.78) | 138 (9.1) |
|  | North | 4.44 (2.06, 9.26) | 0.19 (0.10, 0.40) | 4.59 (2.19, 9.28) | 1.86 (1.49, 2.29) | 87 (5.8) |
|  | West | 3.11 (1.47, 6.05) | 0.17 (0.08, 0.35) | 5.49 (2.86, 10.39) | 2.66 (2.27, 3.09) | 171 (11.3) |
|  | South | 1.93 (0.98, 3.76) | 0.54 (0.29, 1.16) | 1.54 (0.73, 3.07) | 0.60 (0.43, 0.78) | 162 (10.7) |
|  | Southwest | 4.17 (1.65, 9.82) | 0.27 (0.08, 0.74) | 3.33 (1.35, 8.08) | 1.00 (0.75, 1.29) | 31 (2.0) |

Modern methods=Modern contraceptive methods; Media exposure represents a combination of variables for frequency of reading newspapers or magazines, listening to radio, watching television, and use of internet; CrI=Credible Interval.
