# Supplementary material for: A subnational socioeconomic assessment of family planning levels, projections, and disparities among married women of reproductive age in Cameroon
Source: PLoS One. 2025 Feb 14;20(2):e0318650. doi: 10.1371/journal.pone.0318650 (PMC11828404; doi:10.1371/journal.pone.0318650)
Supplement: S9 Table — (DOCX) [file pone.0318650.s009.docx]

**S9 Table: Differences in posterior means of main and altered (weak priors) models by area of residence**

|  |  | **Posterior mean difference [model with vague vs weak priors for hyperparameters]** | | | | | | | | |
| --- | --- | --- | --- | --- | --- | --- | --- | --- | --- | --- |
| COUNTRY/  Region | Urban (U)/  Rural (R) | Modern contraceptive use | | | Unmet need for modern methods | | | Demand satisfied with modern methods | | |
|  |  | **2000** | **2015** | **2030** | **2000** | **2015** | **2030** | **2000** | **2015** | **2030** |
| CAMEROON | U | 0.02 | -0.02 | 0.02 | -0.03 | -0.04 | -0.04 | -0.05 | -0.10 | -0.05 |
|  | R | -0.01 | 0.00 | -0.04 | -0.03 | -0.02 | -0.03 | -0.05 | -0.03 | -0.09 |
| Adamawa | U | -0.01 | -0.03 | -0.04 | 0.00 | 0.00 | -0.01 | 0.01 | 0.02 | 0.04 |
|  | R | 0.01 | 0.00 | 0.00 | 0.02 | 0.01 | -0.02 | 0.00 | -0.02 | -0.01 |
| Centre | U | 0.08 | 0.08 | 0.10 | -0.03 | -0.03 | 0.01 | -0.02 | -0.03 | -0.01 |
|  | R | -0.04 | -0.08 | -0.13 | -0.05 | -0.04 | 0.02 | -0.05 | -0.07 | -0.06 |
| East | U | 0.05 | 0.06 | 0.08 | 0.01 | 0.03 | -0.06 | -0.03 | -0.02 | -0.01 |
|  | R | -0.02 | -0.04 | -0.08 | 0.02 | 0.01 | -0.07 | -0.01 | -0.02 | -0.01 |
| Far North | U | 0.03 | 0.06 | 0.11 | -0.07 | -0.08 | 0.01 | -0.05 | -0.11 | -0.08 |
|  | R | 0.00 | 0.01 | 0.02 | -0.05 | -0.04 | -0.10 | -0.01 | -0.04 | -0.10 |
| Littoral | U | 0.04 | 0.03 | 0.05 | -0.07 | -0.10 | 0.01 | -0.03 | -0.03 | -0.01 |
|  | R | -0.05 | -0.06 | -0.08 | 0.01 | 0.01 | 0.02 | -0.06 | -0.06 | -0.06 |
| Northwest | U | -0.01 | 0.00 | -0.01 | 0.03 | 0.03 | 0.05 | -0.02 | 0.00 | 0.01 |
|  | R | -0.01 | -0.02 | -0.05 | 0.01 | 0.01 | 0.07 | -0.04 | -0.06 | -0.05 |
| North | U | 0.03 | 0.11 | 0.20 | 0.05 | 0.06 | -0.08 | -0.02 | -0.02 | 0.00 |
|  | R | 0.00 | -0.02 | -0.10 | 0.03 | 0.04 | 0.10 | 0.00 | 0.00 | 0.00 |
| West | U | -0.09 | -0.14 | -0.18 | -0.03 | -0.02 | -0.01 | -0.05 | -0.08 | -0.05 |
|  | R | 0.05 | 0.06 | 0.06 | -0.11 | -0.10 | -0.02 | -0.01 | 0.00 | -0.03 |
| South | U | -0.05 | -0.12 | -0.12 | 0.11 | 0.11 | -0.05 | -0.02 | -0.08 | -0.05 |
|  | R | -0.03 | -0.07 | -0.08 | -0.06 | -0.08 | -0.01 | -0.06 | -0.11 | -0.10 |
| Southwest | U | 0.01 | 0.01 | -0.01 | -0.02 | -0.02 | -0.11 | 0.00 | 0.02 | 0.01 |
|  | R | 0.02 | 0.03 | -0.01 | -0.06 | -0.05 | -0.03 | 0.00 | 0.03 | -0.02 |
| Overall | U | 0.01 | 0.01 | 0.02 | -0.01 | -0.01 | 0.00 | -0.02 | 0.06 | -0.02 |
|  | R | -0.01 | -0.02 | -0.04 | -0.02 | -0.02 | -0.02 | -0.03 | -0.03 | 0.05 |
