# Supplementary material for: A subnational socioeconomic assessment of family planning levels, projections, and disparities among married women of reproductive age in Cameroon
Source: PLoS One. 2025 Feb 14;20(2):e0318650. doi: 10.1371/journal.pone.0318650 (PMC11828404; doi:10.1371/journal.pone.0318650)
Supplement: S11 Table — Q1 = poorest, Q2 = poorer, Q3 = middle, Q4 = richer, and Q5 = richest. (DOCX) [file pone.0318650.s011.docx]

**S11 Table: Differences in posterior means of main and altered (weak priors) models by wealth quintile**

|  |  | **Posterior mean difference [model with vague vs weak priors for hyperparameters]** | | | | | | | | |
| --- | --- | --- | --- | --- | --- | --- | --- | --- | --- | --- |
| COUNTRY/  Region | Wealth quintile | Modern contraceptive use | | | Unmet need for modern methods | | | Demand satisfied with modern methods | | |
|  |  | **2000** | **2015** | **2030** | **2000** | **2015** | **2030** | **2000** | **2015** | **2030** |
| CAMEROON | Q1 | 0.00 | 0.00 | 0.04 | 0.07 | 0.05 | 0.02 | -0.02 | -0.04 | 0.11 |
|  | Q2 | -0.02 | -0.04 | -0.10 | -0.05 | -0.01 | 0.04 | 0.00 | -0.05 | -0.15 |
|  | Q3 | -0.08 | -0.14 | -0.22 | 0.27 | -0.02 | 0.06 | -0.11 | -0.26 | -0.29 |
|  | Q4 | -0.12 | 0.00 | -0.11 | -0.08 | 0.03 | 0.08 | -0.19 | -0.02 | -0.15 |
|  | Q5 | -0.17 | -0.10 | -0.03 | -0.03 | 0.15 | 0.04 | -0.17 | -0.07 | 0.04 |
| Adamawa | Q1 | 0.08 | 0.11 | 0.28 | 0.19 | 0.03 | 0.03 | -0.10 | -0.33 | -0.36 |
|  | Q2 | 0.05 | 0.06 | 0.20 | 0.17 | 0.00 | 0.01 | -0.11 | -0.40 | -0.36 |
|  | Q3 | 0.04 | 0.10 | 0.21 | 0.13 | 0.03 | 0.04 | -0.11 | -0.14 | -0.36 |
|  | Q4 | 0.08 | 0.24 | 0.51 | 0.21 | 0.01 | 0.10 | -0.21 | -0.17 | -0.16 |
|  | Q5 | 0.26 | 0.41 | 0.81 | -0.07 | -0.02 | 0.07 | -0.43 | -0.38 | -0.02 |
| Centre | Q1 | -0.14 | -0.28 | -0.73 | -0.21 | -0.17 | -0.20 | -0.12 | -0.45 | -0.67 |
|  | Q2 | -0.21 | -0.03 | -0.20 | -0.57 | -0.03 | -0.22 | -0.20 | 0.11 | -0.02 |
|  | Q3 | -0.11 | -0.25 | -0.74 | -0.34 | -0.16 | -0.24 | 0.00 | -0.27 | -0.64 |
|  | Q4 | -0.07 | -0.39 | -0.36 | 0.04 | -0.09 | -0.13 | 0.09 | -0.40 | -0.11 |
|  | Q5 | -0.44 | -0.29 | -0.91 | -0.33 | -0.12 | -0.27 | -0.33 | -0.16 | -0.55 |
| East | Q1 | -0.06 | -0.04 | -0.04 | -0.28 | -0.06 | -0.03 | 0.00 | -0.01 | -0.02 |
|  | Q2 | -0.21 | -0.05 | -0.16 | -0.22 | 0.01 | -0.15 | -0.06 | 0.13 | -0.12 |
|  | Q3 | -0.33 | -0.12 | -0.17 | -0.40 | -0.02 | -0.05 | -0.18 | 0.02 | -0.06 |
|  | Q4 | -0.21 | -0.12 | -0.19 | -0.23 | -0.18 | -0.13 | 0.04 | 0.03 | -0.10 |
|  | Q5 | -0.30 | -0.19 | -0.23 | -0.17 | -0.10 | -0.03 | 0.05 | 0.03 | -0.02 |
| Far North | Q1 | -0.01 | -0.03 | 0.00 | 0.22 | -0.05 | 0.06 | 0.02 | -0.02 | 0.22 |
|  | Q2 | -0.01 | 0.01 | -0.06 | 0.20 | 0.09 | 0.00 | 0.04 | 0.16 | 0.02 |
|  | Q3 | 0.03 | 0.03 | -0.17 | 0.41 | 0.16 | 0.03 | 0.19 | 0.28 | -0.13 |
|  | Q4 | -0.06 | 0.06 | 0.04 | -0.07 | 0.20 | 0.05 | -0.02 | 0.35 | 0.38 |
|  | Q5 | 0.18 | 0.33 | 0.04 | 0.26 | 0.22 | 0.09 | 0.49 | 0.76 | 0.26 |
| Littoral | Q1 | 0.08 | -0.04 | -0.02 | 0.38 | 0.27 | 0.21 | 0.20 | 0.03 | 0.10 |
|  | Q2 | -0.03 | 0.01 | 0.02 | 0.30 | 0.25 | 0.23 | 0.02 | 0.10 | 0.16 |
|  | Q3 | 0.15 | 0.06 | 0.05 | 0.57 | 0.35 | 0.51 | 0.35 | 0.26 | 0.28 |
|  | Q4 | 0.11 | 0.19 | -0.03 | 0.40 | 0.21 | 0.09 | 0.29 | 0.54 | 0.11 |
|  | Q5 | 0.08 | -0.10 | 0.11 | 0.31 | 0.22 | 0.07 | 0.28 | 0.00 | 0.39 |
| Northwest | Q1 | 0.14 | 0.01 | 0.14 | 0.21 | 0.11 | 0.13 | 0.27 | -0.01 | 0.27 |
|  | Q2 | -0.09 | -0.03 | 0.00 | 0.00 | 0.06 | 0.07 | -0.22 | -0.11 | -0.03 |
|  | Q3 | 0.11 | 0.15 | 0.01 | 0.12 | 0.03 | -0.03 | 0.21 | 0.32 | 0.10 |
|  | Q4 | -0.06 | 0.07 | -0.20 | 0.04 | 0.13 | -0.02 | -0.08 | 0.10 | -0.24 |
|  | Q5 | 0.20 | 0.07 | -0.13 | -0.06 | 0.07 | 0.05 | 0.26 | 0.12 | -0.11 |
| West | Q1 | 0.01 | 0.00 | 0.02 | 0.21 | 0.03 | 0.10 | 0.06 | 0.03 | 0.11 |
|  | Q2 | 0.00 | -0.01 | -0.02 | -0.01 | -0.02 | 0.06 | 0.00 | -0.05 | -0.04 |
|  | Q3 | 0.00 | 0.03 | 0.03 | -0.05 | 0.09 | 0.04 | 0.00 | 0.14 | 0.08 |
|  | Q4 | 0.02 | -0.03 | 0.26 | 0.04 | 0.15 | 0.06 | 0.02 | -0.10 | 0.33 |
|  | Q5 | 0.18 | 0.13 | 0.08 | 0.20 | 0.07 | 0.02 | 0.27 | 0.22 | 0.08 |
| North | Q1 | -0.04 | 0.08 | 0.00 | -0.21 | -0.01 | -0.15 | -0.17 | 0.06 | -0.16 |
|  | Q2 | -0.04 | 0.20 | 0.05 | -0.10 | 0.10 | -0.06 | -0.13 | 0.36 | -0.01 |
|  | Q3 | 0.02 | -0.01 | -0.09 | 0.11 | -0.02 | -0.04 | -0.07 | -0.16 | -0.34 |
|  | Q4 | -0.17 | 0.05 | 0.36 | -0.01 | 0.04 | 0.02 | -0.31 | -0.02 | 0.41 |
|  | Q5 | -0.05 | 0.06 | 0.13 | -0.01 | -0.01 | 0.01 | -0.18 | -0.05 | 0.01 |
| South | Q1 | -0.02 | -0.06 | -0.09 | -0.07 | 0.01 | -0.01 | 0.07 | -0.05 | -0.07 |
|  | Q2 | -0.27 | -0.02 | -0.12 | -0.22 | 0.01 | -0.01 | -0.34 | 0.03 | -0.18 |
|  | Q3 | -0.25 | 0.07 | -0.02 | -0.17 | -0.18 | -0.07 | -0.28 | 0.19 | 0.00 |
|  | Q4 | -0.15 | -0.15 | 0.00 | -0.32 | -0.02 | -0.04 | -0.12 | -0.22 | 0.12 |
|  | Q5 | -0.33 | -0.35 | -0.48 | -0.23 | -0.09 | -0.09 | -0.31 | -0.51 | -0.64 |
| Southwest | Q1 | -0.43 | -0.09 | -0.49 | -0.32 | -0.15 | -0.16 | -0.70 | -0.21 | -0.85 |
|  | Q2 | -0.30 | -0.11 | -0.10 | -0.54 | -0.29 | -0.10 | -0.56 | -0.30 | -0.29 |
|  | Q3 | -0.08 | -0.43 | -0.48 | -0.30 | -0.45 | -0.30 | -0.15 | -0.81 | -0.88 |
|  | Q4 | -0.37 | -0.18 | -0.42 | -0.47 | -0.19 | -0.22 | -0.54 | -0.32 | -0.70 |
|  | Q5 | -0.51 | -0.61 | -0.12 | -0.50 | -0.22 | -0.09 | -0.73 | -0.96 | -0.25 |
| Overall | Q1 | -0.05 | -0.05 | -0.14 | 1.76 | -1.61 | -0.08 | -0.01 | -0.04 | -0.20 |
|  | Q2 | -0.10 | 0.00 | -0.04 | -0.10 | 0.02 | -0.01 | -0.14 | 0.00 | -0.09 |
|  | Q3 | -0.04 | -0.03 | -0.08 | 0.02 | 0.01 | 0.00 | -0.04 | -0.09 | -0.12 |
|  | Q4 | -0.09 | -0.02 | -0.01 | -0.04 | 0.02 | -0.01 | -0.09 | -0.02 | -0.01 |
|  | Q5 | -0.08 | -0.06 | -0.07 | -0.06 | 0.02 | -0.01 | -0.07 | -0.09 | 0.00 |

Q1=poorest, Q2=poorer, Q3=middle, Q4=richer, and Q5=richest.
