# Supplementary material for: A subnational socioeconomic assessment of family planning levels, projections, and disparities among married women of reproductive age in Cameroon
Source: PLoS One. 2025 Feb 14;20(2):e0318650. doi: 10.1371/journal.pone.0318650 (PMC11828404; doi:10.1371/journal.pone.0318650)
Supplement: S12 Table — HDI=Human Development Index; E1 = none, E2 = primary, E3 = secondary, E4 = higher. (DOCX) [file pone.0318650.s012.docx]

**S12 Table: Differences in posterior means of main and altered (no predictor) models by education level**

|  |  | **Posterior mean difference [model with vs without region-level predictor (HDI)]** | | | | | | | | |
| --- | --- | --- | --- | --- | --- | --- | --- | --- | --- | --- |
| COUNTRY/  Region | Level of education | Modern contraceptive use | | | Unmet need for modern methods | | | Demand satisfied with modern methods | | |
|  |  | **2000** | **2015** | **2030** | **2000** | **2015** | **2030** | **2000** | **2015** | **2030** |
| CAMEROON | E1 | 0.09 | 0.04 | 0.11 | 0.24 | 0.51 | 0.52 | 0.28 | 0.19 | 0.42 |
|  | E2 | 0.39 | 0.19 | 0.39 | 0.25 | 0.59 | 0.56 | 0.64 | 0.42 | 0.61 |
|  | E3 | 0.73 | 0.32 | 0.55 | 0.24 | 0.53 | 0.51 | 0.89 | 0.48 | 0.59 |
|  | E4 | 0.94 | 0.37 | 0.60 | 0.14 | 0.32 | 0.33 | 1.01 | 0.47 | 0.51 |
| Adamawa | E1 | 0.06 | 0.00 | 0.07 | -0.02 | 0.23 | 0.10 | 0.12 | -0.02 | 0.14 |
|  | E2 | 0.25 | 0.03 | 0.29 | -0.02 | 0.26 | 0.12 | 0.35 | -0.03 | 0.26 |
|  | E3 | 0.48 | 0.04 | 0.45 | -0.04 | 0.23 | 0.10 | 0.45 | -0.05 | 0.21 |
|  | E4 | 0.59 | -0.14 | 0.22 | -0.08 | 0.28 | 0.07 | 0.47 | -0.02 | 0.08 |
| Centre | E1 | 0.15 | -0.06 | 0.62 | -0.11 | 0.14 | -0.22 | 0.27 | -0.18 | 0.57 |
|  | E2 | 0.26 | -0.09 | 0.85 | -0.14 | 0.18 | -0.23 | 0.29 | -0.18 | 0.60 |
|  | E3 | 0.42 | -0.13 | 0.91 | -0.13 | 0.17 | -0.23 | 0.37 | -0.20 | 0.50 |
|  | E4 | 0.56 | -0.17 | 0.87 | -0.08 | 0.10 | -0.14 | 0.43 | -0.21 | 0.40 |
| East | E1 | 0.04 | -0.04 | 0.30 | -0.09 | 0.11 | -0.04 | 0.07 | -0.14 | 0.40 |
|  | E2 | 0.07 | -0.08 | 0.43 | -0.11 | 0.14 | -0.05 | 0.07 | -0.15 | 0.38 |
|  | E3 | 0.27 | 0.03 | 0.87 | -0.11 | 0.15 | -0.06 | 0.23 | -0.03 | 0.58 |
|  | E4 | 0.18 | -0.17 | 0.68 | -0.06 | 0.12 | -0.03 | 0.15 | -0.22 | 0.41 |
| Far North | E1 | 0.02 | 0.01 | 0.03 | -0.06 | 0.13 | 0.10 | 0.07 | 0.06 | 0.18 |
|  | E2 | 0.14 | 0.07 | 0.22 | -0.06 | 0.14 | 0.11 | 0.36 | 0.24 | 0.46 |
|  | E3 | 0.35 | 0.12 | 0.40 | -0.07 | 0.15 | 0.11 | 0.48 | 0.23 | 0.34 |
|  | E4 | 0.34 | 0.03 | 0.36 | -0.08 | 0.08 | 0.08 | 0.31 | 0.21 | 0.47 |
| Littoral | E1 | -0.10 | -0.47 | -0.41 | -0.62 | -0.40 | -0.54 | -0.28 | -0.86 | -0.77 |
|  | E2 | -0.07 | -0.43 | -0.37 | -0.55 | -0.35 | -0.50 | -0.22 | -0.75 | -0.67 |
|  | E3 | -0.14 | -0.66 | -0.57 | -0.46 | -0.31 | -0.49 | -0.31 | -0.91 | -0.80 |
|  | E4 | -0.17 | -0.91 | -0.73 | -0.27 | -0.21 | -0.33 | -0.34 | -0.94 | -0.72 |
| Northwest | E1 | 0.07 | -0.09 | -0.15 | -0.20 | 0.04 | 0.01 | 0.09 | -0.22 | -0.32 |
|  | E2 | 0.12 | -0.14 | -0.19 | -0.17 | 0.03 | 0.03 | 0.14 | -0.26 | -0.30 |
|  | E3 | 0.21 | -0.24 | -0.29 | -0.13 | 0.02 | 0.01 | 0.17 | -0.31 | -0.32 |
|  | E4 | 0.17 | -0.39 | -0.40 | -0.15 | 0.04 | 0.05 | 0.15 | -0.34 | -0.29 |
| North | E1 | 0.03 | 0.01 | 0.07 | 0.00 | 0.33 | 0.27 | 0.07 | 0.01 | 0.25 |
|  | E2 | 0.07 | -0.01 | 0.14 | -0.01 | 0.32 | 0.28 | 0.12 | -0.05 | 0.28 |
|  | E3 | 0.50 | 0.12 | 0.65 | 0.00 | 0.29 | 0.24 | 0.46 | 0.06 | 0.28 |
|  | E4 | 0.61 | 0.08 | 0.62 | -0.02 | 0.05 | 0.04 | 0.51 | 0.16 | 0.42 |
| West | E1 | -0.01 | -0.20 | -0.29 | -0.25 | -0.05 | -0.04 | -0.07 | -0.56 | -0.76 |
|  | E2 | 0.02 | -0.33 | -0.43 | -0.36 | -0.08 | -0.03 | -0.03 | -0.57 | -0.67 |
|  | E3 | 0.04 | -0.58 | -0.77 | -0.27 | -0.04 | -0.04 | -0.03 | -0.65 | -0.71 |
|  | E4 | -0.06 | -0.92 | -1.01 | -0.32 | -0.11 | -0.08 | -0.13 | -0.80 | -0.67 |
| South | E1 | -0.03 | -0.10 | -0.02 | -0.33 | -0.25 | -0.56 | -0.13 | -0.33 | -0.07 |
|  | E2 | -0.07 | -0.37 | -0.01 | -0.59 | -0.43 | -0.87 | -0.12 | -0.52 | -0.05 |
|  | E3 | -0.13 | -0.49 | -0.04 | -0.61 | -0.43 | -0.89 | -0.19 | -0.57 | -0.10 |
|  | E4 | -0.26 | -0.78 | -0.19 | -0.38 | -0.29 | -0.67 | -0.29 | -0.81 | -0.23 |
| Southwest | E1 | -0.03 | -0.59 | -1.04 | -0.30 | -0.14 | -0.08 | -0.30 | -0.77 | -0.51 |
|  | E2 | 0.02 | -0.42 | -0.87 | -0.51 | -0.26 | -0.17 | -0.14 | -0.76 | -0.87 |
|  | E3 | 0.02 | -0.46 | -0.90 | -0.41 | -0.20 | -0.12 | -0.14 | -0.75 | -0.85 |
|  | E4 | -0.09 | -0.73 | -1.18 | -0.48 | -0.20 | -0.14 | -0.33 | -0.86 | -0.69 |
| Overall | E1 | 0.03 | -0.14 | -0.06 | -0.16 | 0.06 | -0.04 | 0.02 | -0.26 | -0.04 |
|  | E2 | 0.11 | -0.14 | 0.04 | -0.21 | 0.05 | -0.07 | 0.13 | -0.24 | 0.00 |
|  | E3 | 0.25 | -0.18 | 0.11 | -0.18 | 0.05 | -0.08 | 0.21 | -0.24 | -0.03 |
|  | E4 | 0.26 | -0.34 | -0.01 | -0.16 | 0.02 | -0.07 | 0.18 | -0.31 | -0.03 |

HDI=Human Development Index; E1=none, E2=primary, E3=secondary, E4=higher.
