# Supplementary material for: A subnational socioeconomic assessment of family planning levels, projections, and disparities among married women of reproductive age in Cameroon
Source: PLoS One. 2025 Feb 14;20(2):e0318650. doi: 10.1371/journal.pone.0318650 (PMC11828404; doi:10.1371/journal.pone.0318650)
Supplement: S1 Appendix — (DOCX) [file pone.0318650.s014.docx]

**S1 Appendix**

**S-Methods: Bayesian analysis**

The Bayesian approach was selected due to the aim to generate probability over frequentist estimates. The Bayesian methodologies are favoured for small-area assessments [1, 2] such as ours due to substantial variabilities that exist in trends across sub-regions within a country [2, 3] and increased data uncertainty [1]. Unlike with the frequentist [2, 3], this approach allows via the use of probability to more broadly incorporate both sampling and other kinds of uncertainty in estimations. Still, the main advantage here is the generation of probabilistic-oriented inferences which are necessary especially around future values.

***S-Methods 1: Trends model***

The following Bayesian hierarchical regression model was used to estimate family planning indicators for each socioeconomic category (SEC) within each region of Cameroon;

$$y_{ijkl} \sim Normal(\hat{y}_{jkl},\tau^{2})$$

$$\hat{y}_{jkl}= \beta_{0, jk}+ \beta_{1, jk}{year}_{jk}+ \beta_{2,jk}{HDI}_{jk}+ Z_{1jk}+ Z_{2ik}$$

$$\beta_{jk}\sim N(\beta_{j},\sigma_{J}^{2})$$

$$\beta_{j}\sim N(\beta,\sigma^{2})$$

$$\beta\sim Normal(0, {10}^{2})$$

$$\sigma_{j},\sigma\sim Gamma(0.01, 0.01)$$

$$\tau\sim Uniform(0,{10}^{2})$$

Where, $\hat{y}_{jkl}$ is the logit-transformed probability of either use of, unmet need for, or demand satisfied with modern methods for the *j*:th socioeconomic category (SEC) (i.e., wealth, education, or area of residence), in the *k*:th region, and the *l*:th year. Human Development Index (HDI) were the region (*k*) and year-specific (*l*) predictor variables for each socioeconomic category *j*. $\beta_{jk}=[\beta_{0,jk}, \beta_{1, jk}{, \beta}_{2,jk,}]$ is the SEC-specific linear regression model parameter vector which includes intercept ($\beta_{0,jk}$) and the slopes for year ($\beta_{1, jk}$) and HDI ($\beta_{2,jk,}$).$\tau^{2}$ is the model error variance, $\beta_{k}$=[$\beta_{0,k},\beta_{1,k},\beta_{2,k}]$ is the vector of model parameter mean for region *k*. . $Z_{jk}$ and $Z_{ik}$ are the random components defined for SEC within regions, and regions per year, respectively. $\sigma_{k}^{2}=[\sigma_{0,k}^{2}{, \sigma}_{1,k}^{2},\sigma_{2,k}^{2}]$ is the vector of variance of model parameters among SECs belongings to region *k*, while $\beta=[\beta_{0}, \beta_{1}{, \beta}_{2,}]$ and $\sigma^{2}=[\sigma_{0}^{2}{,\sigma}_{1}^{2}, \sigma_{2}^{2}]$ are the means and variance among regions, respectively.

Vaguely-informative prior distributions were assigned to $\beta,\tau,\sigma_{j}, and \sigma$ which represent the hyperparameters follow a normal distribution with mean 0 and variance 100; a uniform distribution with lower (0) and upper (100) limits; and a gamma distribution with shape parameter k(0.01) and scale parameter ($\theta)$(0.01), respectively. The hyperparameters of $\tau,\sigma_{j}, and \sigma$ $\tau, \sigma_{i}, and \sigma$are considered non-informative as there is no information about their distribution.

***S-Methods 2: Sensitivity analysis***

Check for sensitivity of results for of the three family planning indicators was examined via (1) the exclusion of region-level covariate [Human Development Index (HDI)], and (2) altering priors for the hyperparameters.

(1) Exclusion of region-level covariate:

In line with preceding literature [4, 5], HDI was excluded from a rerun of the model to assess the role of the region-level covariate. Differences between mean results for the two model sets (i.e., with and without HDI) were then compared.

(2) Altering priors for the hyperparameters:

Here, hyperparameters were assigned weakly informative instead of vaguely-informative prior (hyper-prior) distributions specified in the main model [6]. Hyperparameters are shared by all intercept coefficients to borrow strength and facilitate parameters smoothing from each group [7, 8]. Thus, in assigning hyperparameters, there are benefits of complete-pooling and no-pooling for the model. In our main model, priors for $\beta(\beta_{0}, \beta_{1}, \beta_{2})$ was assigned a normal distribution with mean = zero and standard deviation = 100, $\beta_{0}, \beta_{1}, \beta_{2}\sim N(0, 100)$. For hyper-priors, both $Z_{1}$and $Z_{2}$ were assigned normal distributions with mean = zero and standard deviations = $\sigma_{1}^{2}$ (with inverse-gamma distribution) and100, respectively. Gelman *et al*., (2006), advocate for proper but weak prior distribution, since separation is rarely an issue in the case of linear regression [9]. Nonetheless, we check for collinearity since this is an issue that still could arise. For this sensitivity analysis, considering that hyperparameter have some influences on all intercept coefficients, the half-Cauchy distribution, a weekly informative prior for hyperparameters ($\tau, \sigma_{j}, and \sigma$) ~ half-Cauchy (0, 25) was applied. After altering prior distribution, the mean absolute differences between the two sets of results were also compared.

***S-Methods 3: Determinants analysis***

The determinants analysis of each family planning indicator was conducted following a Bayesian hierarchical model. For this three-level logistic regression, random intercepts were defined individuals nested within households and households (HHs) within enumeration area/community or primary sampling units (PSUs). These specifications allow for for dependency across the data hierarchy unlike in classical regression models where individuals predictors will be independent of the group effects across HHs and PSUs. Based on standard assumption of a Bernoulli distribution, the log of the probability [10] of either modern contraceptive use, unmet need for, or demand satisfied with modern methods of family planning was modeled as follows:

$$y_{ijk} \sim Bern\left( p_{ijk} \right)$$

$$logit(p_{ijk})=\log\left[ \frac{p_{ijk}}{1-p_{ijk}} \right]=\beta_{0jk}+\beta_{1}X_{ijk}+\beta_{2}Z_{ijk}+u_{0jk}$$

where $p_{ij}$ is the probability of the binary outcome (modern contraceptive use, unmet need for, or demand satisfied with modern methods) for women *i* in household *j* in community *k*. The intercept $\beta_{0jk}$ is assumed to vary randomly. $\beta_{1}$ and $\beta_{2}$ refer to slopes of individual- ($X_{ijk}$) and community-level ($Z_{ijk}$) predictors, respectively. $u_{0jk}$ represents the random part of the model. Default normal priors for regression cofficients, $\beta\sim N(0, 100)$ and inverse-gamma prior for the variance components,$\sigma\sim inverse-gamma(0.01, 0.01)$.

**Extended References**

1. Alexander M, Alkema L. A Bayesian hierarchical model to estimate subnational populations of women of reproductive age. Paper presented at PAA 2018. 2018.

2. Bryant JR, Graham PJ. Bayesian Demographic Accounts: Subnational Population Estimation Using Multiple Data Sources. Bayesian Analysis. 2013;8(3):International Society for Bayesian Analysis: 591–622.

3. Wilson TG, Bell M. Probabilistic regional population forecasts: The example of Queensland, Australia. Geographical Analysis. 2008;39(1).

4. Rahman MM, Taniguchi H, Nsashiyi RS, Islam R, Mahmud SR, Rahman S, et al. Trend and projection of skilled birth attendants and institutional delivery coverage for adolescents in 54 low- and middle-income countries, 2000-2030. BMC medicine. 2022;20(1):46. Epub 2022/02/05. doi: 10.1186/s12916-022-02255-x. PubMed PMID: 35115000; PubMed Central PMCID: PMCPMC8813474.

5. Nsashiyi RS, Rahman MM, Ndam LM, Hashizume M. Exploiting the Bayesian approach to derive counts of married women of reproductive age across Cameroon for healthcare planning, 2000-2030. Sci Rep. 2022;12(1):18075. Epub 2022/10/28. doi: 10.1038/s41598-022-23089-w. PubMed PMID: 36302837; PubMed Central PMCID: PMCPMC9613669.

6. Gelman A, Jakulin A, Pittau MG, Su Y-S. A weakly informative default prior distribution for logistic and other regression models. The annals of applied statistics. 2008;2(4):1360-83.

7. Ntzoufras I. Bayesian modeling using WinBUGS: John Wiley & Sons; 2011.

8. Gelman A. Prior distributions for variance parameters in hierarchical models (comment on article by Browne and Draper). Bayesian analysis. 2006;1(3):515-34.

9. Gelman A. Prior Distributions for Variance Parameters in Hierarchical Models. Bayesian Analysis. 2006;1. doi: 10.1214/06-BA117A.

10. Gelman A, Carlin JB, Stern HS, Dunson D, Vehtari A, Rubin D. Bayesian data analysis, third edition2013. 1-646 p.
