# Supplementary material for: A subnational socioeconomic assessment of family planning levels, projections, and disparities among married women of reproductive age in Cameroon
Source: PLoS One. 2025 Feb 14;20(2):e0318650. doi: 10.1371/journal.pone.0318650 (PMC11828404; doi:10.1371/journal.pone.0318650)
Supplement: Attachment — Submitted filename: PONE-D-24-06584 MEslami 20240911.pdf [file pone.0318650.s015.pdf]

PLOS ONE

The Editor-in-Chief, PLOS ONE journal,

A subnational socioeconomic assessment of family planning levels, projections, and disparities among married women of reproductive age in Cameroon

--Manuscript Draft--

**Manuscript Number:**	PONE-D-24-06584
**Article Type:**	Research Article
**Full Title:**	The Editor-in-Chief, PLOS ONE journal,<br><br>A subnational socioeconomic assessment of family planning levels, projections, and disparities among married women of reproductive age in Cameroon
**Short Title:**	A socioeconomic assessment of family planning across regions of Cameroon
**Corresponding Author:**	Raïssa Shiyghan Nsashiyi, PhD<br>Institute for Nature, Health, and Agricultural Research (INHAR)<br>Buea, Southwest CAMEROON
**Keywords:**	Reproductive health, Family Planning, Contraceptive, Unmet Need, Demand Satisfied, Socioeconomic, Subnational
**Abstract:**	Abstract<br><br>Background: Local-level socioeconomic gradients can drive inequities in access to reproductive health services/commodities particularly in developing countries. Because previous studies mainly focus on national-level disparities, we evaluate performance across Cameroon's subnational territories. Furthermore, we incorporate HIV status (a key but largely omitted covariate) in our assessment of determinants.<br>Methods: A Bayesian hierarchical model incorporated with Cameroon Demographic and Health Survey cross-sectional data (between 1991 and 2018) was employed to generate estimates of family planning indicators among married women of reproductive age per residence, wealth, and education categories within each region. Slope index of inequality was used to quantify disparities. The determinants analysis involved Bayesian logistic regression.<br>Results: Estimates for 2023 revealed that the Centre region's urban and rural areas had the highest modern contraceptive prevalence rate overall with 49.0% (24.9–73.8) and 28.2% (12.4–52.3), respectively. The rural Far North had the least estimate [3.9% (1.5–10.5)]. Demand satisfied with modern methods was highest among Adamawa region's richest quintile [82.9% (58.1 to 94.4)] and higher educated [85.9% (69.5 to 94.2)], and lowest among the East region's poorest [5.3% (1.5 to 16.5)] and Far North's none-educated [8.6% (3.3 to 20.4)]. Unmet need for modern methods was lowest among the West region's richest [5.1% (1.8 to 13.5)] and highest among the Littoral's poorest [23.1% (9.4 to 47.4)]. 2030 projections show the widest wealth- and education-based gaps for demand satisfied with modern methods in the Adamawa [27.0 percentage points (%p) (2.3 to 51.6) and 79.3%p (73.9 to 84.7), respectively]. Age ≥ 20 years, higher education level, practising Catholic/Christian religion, having ≥ one living child(ren), and higher household wealth quintile, were associated with increased odds of modern contraceptive use.<br>Conclusion: Even though the demand for family planning is highly diverse, greater attention is needed among rural, poorer, less educated populations, especially in the Northern regions.
**Order of Authors:**	Raïssa Shiyghan Nsashiyi, PhD
	Md Mizanur Rahman, PhD
	Lawrence Monah Ndam, PhD
	Masahiro Hashizume, PhD
**Additional Information:**	
**Question**	**Response**

Powered by Editorial Manager® and ProduXion Manager® from Aries Systems Corporation

**Financial Disclosure**<br><br>Enter a financial disclosure statement that describes the sources of funding for the work included in this submission. Review the submission guidelines for detailed requirements. View published research articles from *PLOS ONE* for specific examples.<br><br>This statement is required for submission and **will appear in the published article** if the submission is accepted. Please make sure it is accurate.<br><br>**Funded studies**<br>Enter a statement with the following details:<br>• Initials of the authors who received each award<br>• Grant numbers awarded to each author<br>• The full name of each funder<br>• URL of each funder website<br>• Did the sponsors or funders play any role in the study design, data collection and analysis, decision to publish, or preparation of the manuscript?<br><br>Did you receive funding for this work?	The author(s) received no specific funding for this work.
**Competing Interests**<br><br>Use the instructions below to enter a competing interest statement for this submission. On behalf of all authors, disclose any competing interests that could be perceived to bias this work—acknowledging all financial support and any other relevant financial or non-financial competing interests.<br><br>This statement is required for submission and **will appear in the published article** if the submission is accepted. Please make sure it is accurate and that any funding sources listed in your Funding Information later in the submission form are also declared in your Financial Disclosure statement.	The authors have declared that no competing interests exist.

View published research articles from *PLOS ONE* for specific examples.<br><br>**NO authors have competing interests**<br><br>Enter: *The authors have declared that no competing interests exist.*<br><br>**Authors with competing interests**<br><br>Enter competing interest details beginning with this statement:<br><br>*I have read the journal's policy and the authors of this manuscript have the following competing interests: [insert competing interests here]*<br><br>* typeset	
**Ethics Statement**<br><br>Enter an ethics statement for this submission. This statement is required if the study involved:<br><br>• Human participants<br>• Human specimens or tissue<br>• Vertebrate animals or cephalopods<br>• Vertebrate embryos or tissues<br>• Field research<br><br>Write "N/A" if the submission does not require an ethics statement.<br><br>General guidance is provided below. Consult the submission guidelines for detailed instructions. **Make sure that all information entered here is included in the Methods section of the manuscript.**	N/A

Powered by Editorial Manager® and ProduXion Manager® from Aries Systems Corporation

**Format for specific study types**<br><br>**Human Subject Research (involving human participants and/or tissue)**<br>• Give the name of the institutional review board or ethics committee that approved the study<br>• Include the approval number and/or a statement indicating approval of this research<br>• Indicate the form of consent obtained (written/oral) or the reason that consent was not obtained (e.g. the data were analyzed anonymously)<br><br>**Animal Research (involving vertebrate animals, embryos or tissues)**<br>• Provide the name of the Institutional Animal Care and Use Committee (IACUC) or other relevant ethics board that reviewed the study protocol, and indicate whether they approved this research or granted a formal waiver of ethical approval<br>• Include an approval number if one was obtained<br>• If the study involved *non-human primates*, add *additional details* about animal welfare and steps taken to ameliorate suffering<br>• If anesthesia, euthanasia, or any kind of animal sacrifice is part of the study, include briefly which substances and/or methods were applied<br><br>**Field Research**<br><br>Include the following details if this study involves the collection of plant, animal, or other materials from a natural setting:<br>• Field permit number<br>• Name of the institution or relevant body that granted permission	
**Data Availability**<br><br>Authors are required to make all data underlying the findings described fully available, without restriction, and from the time of publication. PLOS allows rare exceptions to address legal and ethical concerns. See the PLOS Data Policy and FAQ for detailed information.	Yes - all data are fully available without restriction

A Data Availability Statement describing where the data can be found is required at submission. Your answers to this question constitute the Data Availability Statement and **will be published in the article**, if accepted.<br><br>**Important:** Stating 'data available on request from the author' is not sufficient. If your data are only available upon request, select 'No' for the first question and explain your exceptional situation in the text box.<br><br>Do the authors confirm that all data underlying the findings described in their manuscript are fully available without restriction?	
**Describe where the data may be found in full sentences. If you are copying our sample text, replace any instances of XXX with the appropriate details.**<br><br>• If the data are **held or will be held in a public repository**, include URLs, accession numbers or DOIs. If this information will only be available after acceptance, indicate this by ticking the box below. For example: *All XXX files are available from the XXX database (accession number(s) XXX, XXX.).*<br>• If the data are all contained **within the manuscript and/or Supporting Information files**, enter the following: *All relevant data are within the manuscript and its Supporting Information files.*<br>• If neither of these applies but you are able to provide **details of access elsewhere**, with or without limitations, please do so. For example:<br><br>*Data cannot be shared publicly because of [XXX]. Data are available from the XXX Institutional Data Access / Ethics Committee (contact via XXX) for researchers who meet the criteria for access to confidential data.*<br><br>*The data underlying the results presented in the study are available from (include the name of the third party*	The data used in this study is freely available upon request at the Demographic and Health Survey Program website: https://dhsprogram.com/data/available-datasets.cfm?ctryid=4

and contact information or URL).<br>• This text is appropriate if the data are owned by a third party and authors do not have permission to share the data.<br><br>* typeset	
Additional data availability information:	

Manuscript Click here to access/download;Manuscript;Manuscript.docx

Title

A subnational socioeconomic assessment of family planning levels, projections, and disparities among married women of reproductive age in Cameroon

Raïssa Shiyghan Nsashiyi (PhD)[1*], Md Mizanur Rahman (PhD)[2], Lawrence Monah Ndam (PhD)[1,3], Masahiro Hashizume (PhD)[4]

[1]Institute for Nature, Health, and Agricultural Research (INHAR), P.O Box 71 Buea, Southwest Region, Cameroon.

[2]Research Centre for Health Policy and Economics, Hitotsubashi Institute for Advanced Study, Hitotsubashi University, 2-1 Naka, Kunitachi Tokyo 186-8601, Japan.

[3]Agroecology Laboratory, Department of Agronomic and Applied Molecular Sciences, Faculty of Agriculture and Veterinary Medicine, University of Buea, P.O Box 63 Buea, Southwest Region, Cameroon.

[4]Department of Global Health Policy, Graduate School of Medicine, The University of Tokyo, 7-3-1, Hongo, Bunkyo-ku, Tokyo, Japan

* Corresponding author

E-mail: raissa.nsashiyi@gmail.com (RSN)

Abstract

Background: Local-level socioeconomic gradients can drive inequities in access to reproductive health services/commodities particularly in developing countries. Because previous studies mainly focus on national-level disparities, we evaluate performance across Cameroon's subnational territories. Furthermore, we incorporate HIV status (a key but largely omitted covariate) in our assessment of determinants.

Methods: A Bayesian hierarchical model incorporated with Cameroon Demographic and Health Survey cross-sectional data (between 1991 and 2018) was employed to generate estimates of family planning indicators among married women of reproductive age per residence, wealth, and education categories within each region. Slope index of inequality was used to quantify disparities. The determinants analysis involved Bayesian logistic regression.

Results: Estimates for 2023 revealed that the *Centre* region's urban and rural areas had the highest modern contraceptive prevalence rate overall with 49.0% (24.9–73.8) and 28.2% (12.4–52.3), respectively. The rural *Far North* had the least estimate [3.9% (1.5–10.5)]. Demand satisfied with modern methods was highest among *Adamawa* region's richest quintile [82.9% (58.1 to 94.4)] and higher educated [85.9% (69.5 to 94.2)], and lowest among the *East* region's poorest [5.3% (1.5 to 16.5)] and *Far North*'s none-educated [8.6% (3.3 to 20.4)]. Unmet need for modern methods was lowest among the *West* region's richest [5.1% (1.8 to 13.5)] and highest among the *Littoral*'s poorest [23.1% (9.4 to 47.4)]. 2030 projections show the widest wealth- and education-based gaps for demand satisfied with modern methods in the *Adamawa* [27.0 percentage points (%p) (2.3 to 51.6) and 79.3%p (73.9 to 84.7), respectively]. Age ≥ 20 years, higher education level, practising Catholic/Christian religion, having ≥ one living child(ren), and higher household wealth quintile, were associated with increased odds of modern contraceptive use.

Conclusion: Even though the demand for family planning is highly diverse, greater attention is needed among rural, poorer, less educated populations, especially in the *Northern* regions.

Introduction

Given the emphasis on enhancing equity in the Sustainable Development Goal (SDG) era [1], it is crucial that current assessments, including the SDG 3.7.1 focus on the demand for family planning that is satisfied with modern methods, reflect progress relating to inequities that distinguished levels in the past [2]. Despite renewed efforts [3] and gains made in the expansion of access to basic family planning services and commodities across low- and middle-income countries (LMICs) [4, 5], evidence continues to mount of sustained inequities that are defined by varied socioeconomic characteristics [2, 6].

Disparities have persisted such that the more disadvantaged socioeconomic groups have less coverage for family planning [2, 6], even as they bear higher risks for adverse health outcomes (like maternal and child mortality) that these services have been shown to prevent [7]. Studies from LMICs show lower levels of use of [8] and demand satisfied with modern contraceptive methods [9] among poorer, uneducated, and rural populations of women who are married or in a union. For Cameroon in particular, the wealth-, education-, and area of residence-based gaps in demand satisfied with modern methods have not only persisted but also widened by at least 16.3, 15.6, and 8.2%, respectively, between 1991 and 2011 [6].

Having recorded an overall increase in modern contraceptive use [5] amidst recent increases in investments toward expanding access to family planning services nationwide [10], the reported gaps indicate the need for investigations to further guide specific strategies. Previous studies conducted in LMICs mainly focus on national-level socioeconomic disparities [6, 11-14]. However, further disaggregated analyses by smaller areas can be particularly useful for the implementation of programmes per subpopulation needs. In Cameroon, subnational socioeconomic assessments of family planning performance could inform policies and facilitate targeted interventions for disadvantaged groups, at lower levels that align with the country's administratively organized healthcare delivery system [15].

Meanwhile, a gap exists in the assessment of determinants of family planning where HIV status is incorporated as a covariate. Previous studies conducted across sub-Saharan African (SSA) countries often omit this variable [16-22] despite the region having the highest infection rates globally [23], and infection status influencing the use of common barrier contraceptives like

male/female condoms [24, 25]. Aside its role in family planning for prevention of unintended pregnancy, condoms offer protection against the transmission of HIV and other sexually transmitted infections [24, 25]. More so, previous evidence shows HIV-positive status to be a significant predictor of family planning practice in general [26].

This study, therefore, aims to explore area of residence-, wealth-, and education-based levels of and disparities in modern contraceptive use, unmet need for, and demand satisfied with modern methods across Cameroon's 10 regions up to 2030. Additionally, individual- and -community-level socio-demographic and -economic factors that distinguish use and non-use of modern methods of family planning at a national scale are examined.

Methods

Data

Data were sourced from five Demographic Health Survey (DHS) cross-sectional surveys conducted in Cameroon in 1991, 1998, 2004, 2011, and 2018. Sample sizes ranged between 3,871 (DHS-1991) and 1,5426 (DHS-2011). For area of residence-, wealth-, education-based analysis of either family planning indicators, totals of 108, 273, and 213 survey observations were incorporated (Supplementary material, S1 Table).

Outcome variables

Outcome variables were defined in line with previous studies [5, 27, 28]. Modern contraceptive prevalence rate equals the percentage of women who are or whose partners are currently using any modern contraceptive method. Unmet need for modern methods is the percentage of women who are not currently using any method of contraception to prevent pregnancy but want to space or limit childbearing. Demand satisfied with modern methods shows the percentage of women who want to space or limit childbearing and who use a modern contraceptive method. Detailed calculations are presented as supplementary material (S2 Table). Modern contraceptive methods include sterilisations, oral contraceptive pills, intrauterine devices, injectables, implants, condoms, lactational amenorrhea method, standard days method, emergency contraception, and vaginal barrier methods [29].

Predictor variables

Trends analysis: Year-specific Human Development Index (HDI) [30] for each region was used. The HDI is a summary measure of average achievement in key dimensions of human development, including life expectancy, education, and per capita income [31]. HDIs were projected up to 2030 via linear extrapolation. *Determinants analysis*: Following preceding literature [16-20, 32] individual-level variables, i.e., age, level of education, religion, number of living children, media exposure, and wealth quintile, as well as community-level variables, i.e., area of residence and region were incorporated. Additionally, HIV status was included as an individual-level predictor [26]. Both the disparities and determinants assessments represent estimates for married women of reproductive age, i.e., women aged 15 to 49 years who are married or in a union.

Statistical analysis

Each outcome variable was logit transformed to ensure that the modelled estimates remained within the range of 0 to 100%. Three separate Bayesian models [33] were fitted such that estimates of each family planning indicator would be dependent on the corresponding HDIs. These were incorporated in the hierarchical framework each for area of residence, wealth, or education, within regions, and in a time-series manner that would capture changes over time. Final estimates of each family planning indicator are the means that were derived from samples of the posterior distribution via the Markov chain Monte Carlo (MCMC) algorithm. 95% Credible Intervals (CrI) are the 2.5 and 97.5 percentiles of the MCMC samples. Model checks included trace plots for convergence of MCMC and Gelman-Rubin diagnostic statistics [34]. 10,000 iterations with three chains, 10 thinning, and 3000 sample burn-in were used in the MCMC algorithm. A dual approach was used to examine the sensitivity of the results. First, by the exclusion of region-level covariate (i.e., HDI), and second, by the alteration of priors for the hyperparameters. A breakdown of the sensitivity analysis is presented as supplementary information (S1 Appendix, S-methods 2). The slope index of inequality (SII) was employed to assess the magnitude of wealth- and education-based disparities for all three indicators for the years 2015 and 2030. The SII shows the absolute difference between the most-advantaged and most-disadvantaged socioeconomic categories in terms of estimated favourable family planning indicators, and the reverse for the adverse indicator (i.e., unmet need). SIIs range between -100 to +100 where 0 indicates no difference [35].

For the determinants analysis, Bayesian hierarchical logistic regression models was used with random intercepts. The multilevel framework accounts for dependency across the multilevel data, i.e. individuals nested within households, households within communities. The final model selection was based on the deviance information criteria (DIC) comparisons of the relative goodness-of-fit. Further details are presented in the supplemental file (S1 Appendix, S-methods 3). All statistical analysis was conducted with STATA version 17 (Stata Corp, College Station, TX, USA).

Results

Coverage/disparities by area of residence

Fig 1 shows that urban and rural levels of modern contraceptive prevalence and demand satisfied with modern methods steadily increased among married women of reproductive age in Cameroon from 1990 onwards. However, the urban coverage of both indicators has been notably higher compared to the rural coverage. Urban levels of modern contraceptive prevalence were estimated at 29.6% (95% CrI13.0 to 54.4) compared to rural levels of 12.6% (4.9 to 28.8) in 2023. Even though, the gap could further widen as projections show that urban and rural coverage could increase to 36.9% (17.1 to 61.7) and 16.4% (6.5 to 35.6), respectively, in 2030 (Table 1). As for the unmet need for modern methods, there are no clear patterns of changes over time, and urban levels appear to be only slightly lower than the rural. The unmet need for modern methods in urban and rural areas was estimated at 21.4% (13.6 to 31.9) and 22.2% (14.0 to 33.0) in 2023 and projected to 21.7% (13.7 to 32.5) and 22.4% (14.3 to 33.4) in 2030, respectively.

Table 1: Urban and rural estimates of modern contraceptive use for Cameroon and its regions, 2000, 2015, and 2030

**COUNTRY** Region	**Urban(U)/ Rural(R)**	**Modern contraceptive prevalence rate**		
		**2000**	**2015**	**2030**
**CAMEROON**	U	13.8 (5.5–30.8)	21.5 (9.2–43.8)	36.9 (17.1–61.7)
	R	5.1 (1.9–13.2)	8.7 (3.2–21.0)	16.4 (6.5–35.6)
Adamawa	U	8.2 (3.1–20.1)	13.8 (5.4–31.5)	27.4 (11.7–51.5)
	R	1.8 (0.6–4.8)	3.1 (1.1–8.3)	6.9 (2.5–17.0)
Centre	U	19.1 (7.8–40.0)	32.6 (14.7–58.1)	60.7 (35.9–81.9)
	R	8.7 (3.3–21.6)	16.4 (6.7–35.9)	38.3 (18.2–63.3)

East	U	15.0 (6.1–33.1)	25.5 (10.9–49.4)	47.5 (24.3–72.2)
	R	5.4 (2.0–13.7)	9.9 (3.7–23.6)	22.5 (9.4–44.8)
Far North	U	5.1 (1.9–12.9)	17.3 (6.8–37.4)	49.2 (25.7–73.2)
	R	0.5 (0.2–1.2)	1.7 (0.6–4.9)	7.6 (2.8–19.0)
Littoral	U	16.1 (6.5–35.6)	19.1 (7.7–39.1)	27.3 (11.7–51.3)
	R	11.0 (4.3–25.2)	13.3 (5.2–29.5)	19.4 (7.9–40.5)
Northwest	U	14.0 (5.5–31.2)	26.9 (11.4–51.0)	49.7 (26.1–73.7)
	R	8.8 (3.3–21.3)	17.9 (7.5–38.1)	36.9 (17.3–62.0)
North	U	4.4 (1.6–11.7)	17.7 (7.0–38.3)	56.6 (31.7–78.7)
	R	0.4 (0.1–1.1)	1.7 (0.6–4.8)	9.5 (3.5–23.2)
West	U	13.8 (5.3–30.9)	19.3 (7.7–39.8)	30.3 (13.2–55.0)
	R	9.6 (3.7–23.0)	13.7 (5.4–30.8)	22.3 (8.9–45.0)
South	U	19.6 (7.9–40.4)	21.1 (8.6–43.4)	29.8 (12.8–54.1)
	R	11.6 (4.5–26.4)	12.7 (4.9–29.3)	18.7 (7.2–38.5)
Southwest	U	15.4 (5.9–33.6)	22.1 (9.0–44.3)	32.6 (14.7–57.9)
	R	11.2 (4.3–26.2)	16.5 (6.5–35.5)	25.2 (10.7–48.6)

At the region level, the *Centre* recorded the highest of both urban and rural modern contraceptive prevalence of 49.0% (24.9 to 73.8) and 28.2% (12.4 to 52.3), respectively, in 2023. For the urban areas, the *Centre* is followed by the *Northwest* and *East* with modern contraceptive prevalence of 60.7% (35.9 to 81.9), 49.7% (26.1 to 73.7), and 47.5% (24.3 to 72.2), respectively, projected for 2030 (Table 1). The least estimates seen in the rural areas of the *Adamawa*, *Far North*, and *North* should be sustained based on their respective 2030 projections of 6.9% (2.5 to 17.0), 7.6% (2.8 to 19.0), and 9.5% (3.5 to 23.2). All three northern (i.e., *North*, *Far North*, and *Adamawa*) are among the top five with the widest urban-rural gaps in modern contraceptive prevalence. The two others include the *East* and *Centre*. The *North* and *Far North* recorded the most widening gaps, with respective urban versus (vs) rural modern contraceptive prevalence projections of 56.6% (31.7 to 78.7) vs 9.5% (3.5 to 23.2) and 49.2% (25.7 to 73.2) vs 7.6% (2.8 to 19.0) for 2030. This is compared to the estimated 4.4% (1.6 to 11.7) vs 0.4% (0.1 to 1.1) and 5.1% (1.9 to 12.9) vs 0.5% (0.2 to 1.2), respectively, in 2000. The supplementary material (S3 Table) includes urban- and rural estimates of demand satisfied and unmet need for modern methods.

Fig 1. Urban and rural estimates of use of, unmet need, and demand satisfied for modern contraceptive methods in Cameroon, 1990–2030. Lines=Loess fit through median estimates; Circles=Demographic and Health Survey estimates.

Coverage/disparities by wealth status

Fig 2 shows trends in demand satisfied with modern methods per wealth quintile and region of Cameroon. Across the board, levels have remained higher among the richer. In 2023, the highest estimate of 82.9% (58.1 to 94.4) demand satisfied with modern methods was estimated among those in the richest quintile of the *Adamawa* region. This was followed by the richer quintile of *Adamawa* [60.2% (30.2 to 84.0)], richest quintile of the *North* [59.4% (30.1 to 83.9)], and richest quintile of the *Centre* [55.9% (26.9 to 81.3)]. Overall, levels of demand satisfied with modern methods were lower in the poorer wealth quintiles. However, the least estimates included four wealth quintiles in the *East* region, i.e., poorest, poorer, middle, and richer quintile, with 5.3% (1.5 to 16.5), 12.9% (4.1 to 34.5), 14.1% (4.5 to 36.5), and 14.5% (4.6 to 36.4), respectively, in 2023. Notably, only in the *East* were levels shown to decrease over time from 2000 to 2030 across all wealth quintiles. Projections show that the aforementioned top and bottom rankings could be maintained up to 2030. See Fig 2 and the supplemental file (S4 Table). Wealth-based disparities in demand satisfied with modern methods are presented in Table 2. The *Southwest* recorded the narrowest wealth-based gaps, i.e., 9.4 percentage points (%p) (%p) (-12.3 to 31.1) in 2015 and 11.8 %p (-14.0–37.6) projected for 2030. This is closely followed by estimates for the *South* and *Centre*. Even though the *Adamawa* shows the widest gaps, it could also record the largest projected decrease, i.e., from 40.7 %p (10.2 to 71.2) in 2015 to 27.0 %p (2.3 to 51.6) in 2030.

Table 2: Magnitude of socio-economic inequalities in demand satisfied for modern methods for Cameroon and its regions, 2015 and 2030

COUNTRY<br>Region	**Slope Index of Inequality (SII) for demand satisfied with modern methods (%p)**			
	**Wealth-based SII**		**Education-based SII**	
	**2015**	**2030**	**2015**	**2030**
CAMEROON	33.1 (31.8–34.5)	41.9 (40.4–43.3)	56.0 (44.9–67.0)	61.0 (49.2–72.8)
Adamawa	40.7 (10.2–71.2)	27.0 (2.3–51.6)	78.9 (73.7–84.0)	79.3 (73.9–84.7)
Centre	12.6 (-10.6–35.8)	14.9 (-13.6–43.4)	39.1 (29.3–48.8)	34.7 (24.8–44.5)
East	16.9 (10.0–23.8)	15.9 (9.5–22.2)	39.6 (31.1–48.0)	45.0 (33.9–56.0)
Far North	33.4 (16.1–50.7)	42.3 (21.0–63.7)	33.5 (-15.6–82.5)	47.6 (-7.9–99.7)
Littoral	17.3 (13.3–21.3)	20.5 (15.6–25.4)	31.6 (15.6–47.6)	33.5 (16.2–50.9)
Northwest	24.2 (12.9–35.4)	27.9 (13.5–42.4)	24.6 (19.5–29.7)	29.0 (23.5–34.5)
North	37.9 (30.9–44.9)	53.1 (45.8–60.4)	59.2 (32.8–85.5)	70.4 (51.2–89.6)
West	24.0 (18.5–29.5)	29.1 (22.1–36.2)	44.6 (39.5–49.6)	47.8 (41.7–54.0)

South	11.4 (7.8–15.1)	13.6 (9.7–17.5)	35.6 (26.9–44.4)	40.1 (30.5–49.8)
Southwest	9.4 (-12.3–31.1)	11.8 (-14.0–37.6)	-10.0 (-53.5–33.5)	-6.8 (-41.5–28.0)

%p= percentage points.

Fig 2 also includes unmet need for modern methods estimates between 2000 and 2030 across wealth quintiles and regions of Cameroon. Negative trends are observable for each of the quintiles across all regions, and most substantially between 2000 and 2015. Similar to demand satisfied, unmet need for modern methods tends to be more favourable among the richer quintiles. Wherefore, richer quintiles registered lower while poorer quintile showed higher levels of unmet need for modern methods. In 2023, the lowest estimate was registered among the richest in the *West* region [5.1% (1.8 to 13.5)], where according to projections could reach as low as 3.7% (1.3 to 10.0) in 2030. Current (2023) levels are also lower among the richest quintiles in the *East*, *Northwest*, and *South*, poorer and middle quintiles in the *Adamawa*, and poorest quintiles in the *East* and *Far North*. Uniquely, the poorer groups in the *Far North* and *Centre* regions showed lower prevalence of unmet need for modern methods compared to the wealthier. The other end with the highest estimates includes three out of the five quintiles within the *Littoral* region, i.e., the poorest, middle, and poorer quintiles with 23.1% (9.4 to 47.4), 18.6% (7.3 to 40.1), and 17.2% (6.9 to 37.3), respectively, in 2023. Despite the decreases, levels for these three quintiles are to remain the highest with forecasts of at least 15.2% (5.9 to 33.8) (among the poorer) for 2030. Wealth-based estimated values and disparities for all three family planning indicators can be found in supplemental files S4 and S5 Table, respectively.

Fig 2. Demand satisfied and unmet need for modern contraceptive methods by wealth quintile for Cameroon and its regions, 2000, 2015, and 2030.

Coverage/disparities by education level

Estimates of demand satisfied with and unmet need for modern methods by level of education are presented in Fig 3. For demand satisfied with modern methods, except for the *South* region, levels are shown to steadily increase across all educational categories from 2000 to 2030. Like with the wealth-based assessment, those with higher educational attainment tended to record higher levels of demand satisfied with modern methods and vice versa. Levels among those with higher education in *Adamawa* were highest at 85.9% (69.5 to 94.2) in 2023 and 89.5% (76.1 to 95.8) projected for 2030. Oddly, this was followed by those with no education in the *Southwest*

[75.0% (52.2 to 89.2) in 2023] with levels that are higher than in the other groups with higher educational attainment within the same region. Ranked third and fourth are those with higher education in the *Centre* and secondary education in the *North*, respectively. At the bottom with the lowest demand satisfied with modern methods of 8.6% (3.3 to 20.4) in 2023 was the education category 'none' of the *Far North* amongst whom levels are to remain lowest up to 2030 [11.8% (4.7 to 26.5)]. 'None' education groups in the *South* and *Adamawa* closely follow. The education-based disparities in demand satisfied with modern methods (Table 2) were narrowest but negative in the *Southwest* region which has a 2030 projection of -6.8 %p (-41.5–28.0). The largest gaps were seen in the *Adamawa* and *North* regions where the respective magnitudes are estimated to further widen to 79.3%p (73.9 to 84.7) and 70.4%p (51.2 to 89.6) in 2030.

Regarding the unmet need for modern methods, only the *Southwest* and *East* show decreases across all four education levels from 2000 to 2030 (Fig 3). Amidst falling levels, these two regions have the most favourable projections in terms of low unmet need for modern methods, particularly among the 'none' and 'higher' educated, respectively. Lower levels are also projected across all four educational categories in both the *Southwest* and *East* especially compared to the other regions. As of 2023, the least prevalence was among the higher educated in the *North* at 6.4% (3.1 to 12.8), even though levels could slightly rise to 7.3% (3.5 to 14.7) in 2030. At the other end, those with higher education in *Adamawa* showed a noticeably higher unmet need for modern methods, compared to the region's other (lower) education categories as well as overall. Prevalence in this group reached 54.1% (35.3 to 71.7) in 2023 and is projected to slightly increase to 55.0% (36.2 to 72.7) in 2030. Projections are least favourable in the *Far North* due to recording the steepest increases across all educational groups. Supplemental files S5 and S6 Table respectively show disparities and prevalence for each family planning indicator per level of education.

Fig 3. Demand satisfied and unmet need for modern contraceptive methods by level of education for Cameroon and its regions, 2000, 2015, and 2030.

Determinants of family planning

In Table 3, adjusted odds ratios (AORs) from the determinants analysis of modern contraceptive use and unmet need for modern methods among married women of reproductive age in

Cameroon are presented. Individual level variables, age ≥ 20 years, higher education level, practising Catholic or Christian religion, having one or more living child(ren), and higher household wealth quintile, were associated with increased odds of modern contraceptive use, credible intervals inclusive. The odds were higher by 2.23 (1.96 to 2.62) among those aged 20 to 29 years compared to the 15- to 19-year-olds; 2.43 (1.97 to 3.00) among the 'higher' compared to the 'none' educated; 1.38 (1.15 to 1.70) among Catholics relative Muslims; 1.73 (1.39 to 2.14) for those with 4 to 6 relative to no living children; and 3.04 (2.42 to 3.74) among those that lived in households ranked in the richest compared to the poorest quintile. Except in the *South*, living in every other region showed higher odds of modern contraceptive use relative to the *Adamawa*. AORs for unmet need for modern methods mostly reflect the converse of AORs for modern contraceptive use. Crude odds ratios are presented in the supplemental file S7 Table.

There are something which not follow the main trend. Their should be considered in the discussion.

Table 3: Determinants of use of and unmet need for modern methods of family planning in Cameroon, DHS 2018

Variable	Categories	Adjusted Odds Ratio (95% Credible Interval)		Frequency
		Modern contraceptive use	Unmet need for modern methods	
HIV status	Negative	1	1	63
	Positive	0.85 (0.68, 1.03)	1.10 (0.88, 1.38)	1,450
Age group, years	15-19	1	1	117
	20-29	2.23 (1.96, 2.62)	0.42 (0.35, 0.51)	584
	30-39	2.20 (1.84, 2.61)	0.40 (0.33, 0.47)	573
	40-49	1.61 (1.32, 1.95)	0.52 (0.41, 0.67)	239
Level of education	None	1	1	253
	Primary	2.56 (2.08, 3.11)	0.49 (0.38, 0.61)	493
	Secondary	3.11 (2.74, 3.64)	0.35 (0.28, 0.42)	662
	Higher	2.43 (1.97, 3.00)	0.33 (0.26, 0.40)	105
Religion	Muslim	1	1	317
	Catholic	1.38 (1.15, 1.70)	0.77 (0.66, 0.90)	581
	Christian	1.35 (1.15, 1.58)	0.64 (0.57, 0.73)	569
	None/other	0.37 (0.30, 0.45)	1.67 (1.35, 2.09)	46
Living children	None	1	1	37
	1-3	1.32 (1.16, 1.49)	0.97 (0.78, 1.19)	762
	4-6	1.73 (1.39, 2.14)	0.83 (0.72, 0.97)	547
	≥6	1.48 (1.19, 1.85)	1.43 (1.12, 1.79)	167
Media exposure	None	1	1	469
	<Once a week	1.17 (0.96, 1.40)	0.76 (0.67, 0.87)	602
	>Once a week	1.14 (0.94, 1.31)	0.92 (0.76, 1.11)	440
Wealth quintile	Poorest	1	1	192
	Poorer	2.03 (1.83, 2.24)	0.59 (0.46, 0.76)	308

	Middle	2.27 (1.91, 2.67)	0.45 (0.37, 0.55)	383
	Richer	2.07 (1.64, 2.63)	0.48 (0.39, 0.58)	309
	Richest	3.04 (2.42, 3.74)	0.34 (0.29, 0.42)	321
Residence	Rural	1	1	727
	Urban	1.01 (0.82, 1.23)	0.91 (0.77, 1.09)	786
Region	Adamawa	1	1	95
	Centre	1.51 (1.28, 1.76)	0.70 (0.59, 0.82)	318
	East	2.34 (1.85, 2.89)	0.13 (0.10, 0.15)	166
	Far North	2.18 (1.72, 2.70)	0.49 (0.41, 0.59)	132
	Littoral	1.18 (1.02, 1.35)	0.81 (0.67, 0.97)	213
	Northwest	1.92 (1.55, 2.37)	0.60 (0.47, 0.77)	138
	North	1.95 (1.64, 2.34)	0.45 (0.34, 0.60)	87
	West	1.49 (1.29, 1.78)	0.37 (0.30, 0.45)	171
	South	0.93 (0.73, 1.18)	1.56 (1.28, 1.91)	162
	Southwest	1.60 (1.18, 2.18)	0.99 (0.86, 1.18)	31

Modern methods=Modern contraceptive methods; Media exposure represents a combination of variables for frequency of reading newspapers or magazines, listening to radio, watching television, and use of internet.

Sensitivity analysis

The overall absolute differences between the main versus altered models for each family planning indicator and socioeconomic category in 2000, 2015, and 2030 were very minor. Excluding region-level predictors, the differences for use of, unmet need for, and demand satisfied with modern methods respectively ranged between: 0.01 to 0.08%, 0.01 to 0.04%, and 0.00 to 0.07% by area of residence (Supplemental file, S8 Table); 0.00 to 0.03%, 0.00 to 0.05%, and 0.01 to 0.02% by wealth quintile (Supplemental file, S9 Table); and 0.01 to 0.26%, 0.02 to 0.21, and 0.00 to 0.26% by education level (Supplemental file, S10 Table). Altering priors for the hyperparameters, the differences for use of, unmet need for, and demand satisfied with modern methods respectively ranged between: 0.01 to 0.04%, 0.00 to 0.02%, and 0.02 to 0.06% by area of residence (Supplemental file, S11 Table); 0.00 to 0.14%, 0.00 to 1.76%, and 0.00 to 0.20% by wealth quintile (Supplemental file, S12 Table); and 0.06 to 0.15%, 0.01 to 0.05%, and 0.11 to 0.19% by education level (Supplemental file, S13 Table).

Discussion

In this paper, area of residence-, wealth-, and education-based levels, projections of, and disparities in family planning among married women of reproductive age across the 10 regions

of Cameroon were presented. Results for most of the regions revealed higher levels of favourable indicators, i.e., modern contraceptive prevalence and demand satisfied with modern methods, among those in the higher ranked socioeconomic categories such as urban residence, richest wealth quintile, and higher education. For the unfavourable indicator (unmet need for modern methods), estimates mostly reflected the reverse, i.e., higher levels in the lower-ranked socioeconomic categories like rural residence, poorest wealth quintile, and no education.

Across all 10 regions, area of residence-based assessments revealed higher levels of modern contraceptive use in urban compared to rural areas. These findings were not unexpected and they align with disparities recorded from previous surveys conducted at the national level [36]. Studies in other SSA countries [8, 12, 22] have likewise shown noticeably higher urban compared to rural modern contraceptive prevalence at the national level. Similar to the urban trends, all 10 rural settings recorded substantial increases in modern contraceptive prevalence that are projected to continue up to 2030. The *Centre* region stands out because coverage in its rural area is noticeably higher than in the other rural and in 6 out of the 10 urban areas. This wide disparity could be linked to better access to family planning services. Being the country's administrative capital, the *Centre* region has been reported to have the highest share of health resources like personnel, facilities, and health budget per capita [15, 37]. Aside those, HIV rates could be speculated as a factor here given their reported influence on the use of especially barrier contraceptives like male/female condoms [24-26]; and clusters in mainly rural divisions of the *Centre* region (i.e., Haute Sanaga, Nyong et Mfoumou, Nyong et So'o, and Haut Nyong) being recently uncovered among major hot-spots of HIV infection in Cameroon [38]. On the other end, rural areas in the northern regions (*Adamawa*, *Far North*, and *North*) showed substantially lower modern contraceptive prevalence compared to other rural and urban areas. It is worth noting that these three regions each receive much less in terms of health resources per capita compared to the other 7 regions [15, 37]. In the *Far North*, for instance, the per capita health budget and facilities/personnel are, respectively, only one-third and less than half that of the *Centre* [15]. Additionally, given that illiteracy rates are also highest in the aforementioned northern regions (at least 60% each) [39], lack of or limited access to information could be an important contributor, particularly in the rural areas. An important policy focus should thus be to target disparities by area of residence, especially in regions such as the *Far North* and *North* where urban-rural gaps are widening and projected to persist long term.

Previous national-level assessments showed much larger wealth compared to education-related disparities in the demand satisfied with modern methods [6]. However, the regional-level analysis conducted herewith uncovered much wider education- compared to wealth-based gaps, particularly in the northern regions of *Adamawa*, *Far North*, and *North*. Wider disparities across the cited settings may be linked to underlying socioeconomic disadvantages compared to the other regions of the country. For instance, the Human Development Index (a composite measure that incorporates life expectancy, education, and per capita income [31]) for each of the northern regions is less than that for the other regions as well as the national average [30]. In a previous study, the same three regions were found to have the lowest levels of demand satisfied with modern methods in Cameroon [36]. Having shown the widest wealth- and education-based gaps, despite also recording highest estimates among the richer and more educated groups (particularly the *Adamawa* and *North*), this indicates that demand satisfied with modern methods in the [redacted] categories. These findings demonstrate the need to prioritise the poorer and less educated during family planning programming. Meanwhile, for regions with the smallest disparities in demand satisfied with modern methods, only in the *Southwest* did this cut across both the wealth- and education-based assessments. Features that were earlier linked to performances in the other regions such as the socioeconomics, availability of health resources, or relevant health indicators like HIV rate are not distinguish in this region. The *Southwest* historically stands out as an epicenter of higher learning institutions in Cameroon [40]. Whether this transcends to an environment where the less educated have increased odds of an environment with more access to information on issues like contraceptive use is unclear. Nonetheless, community-level assessments are recommended in better-performing regions like *Southwest* to understand policy-applicable elements that can be replicated elsewhere to narrow disparities or improve coverage in family planning demand satisfied among the socioeconomically disadvantaged.

Regarding the unmet need for modern methods, wealth categories across all 10 regions showed positive progress in terms of declining levels and narrowing disparities over time. Importantly, the findings reflect more improvements in the lower-ranked wealth quintiles which is also reflected in the negative SIIs (Supplemental file, S5 Table). Indicating that proportions of married women of reproductive age whose need for birth spacing or limiting is being met using modern contraceptive methods in each region are increasingly from the poorer households. More

unclear are the education-related patterns of unmet need for modern methods, as trends across categories were positive in most regions (6 out of 10), and negative or steady in the rest. Similarly, irregular education-based patterns of unmet need have been reported across regions of Ghana [19]. It is noteworthy that rising levels might not necessarily indicate a regress in performance as trajectories of unmet need are said to largely depend on the contraceptive prevalence level [41]. A multi-country assessment demonstrated that levels of unmet need tend to initially rise until contraceptive prevalence reaches around 20% where an accelerated decrease sets in [41]. Notably, more than half of the education categories across the 10 regions recorded an average modern contraceptive prevalence of less than 20% over the study period. Careful consideration of contraceptive prevalence is therefore required during family planning strategy development where unmet need is among key indicators of performance.

The determinants analysis is relevant in terms of developing comprehensive initiatives aimed at promoting the use of modern contraceptives for family planning. Age, education, wealth, having living children, and religion were deemed as key predictors of either the use or nonuse of modern contraceptives. The findings specifically point toward the need for family planning interventions to prioritise married women of reproductive age who are of teenage age, not/less educated, living in the poorest households, have no living children, and/or practice Islam. Our findings align with prior assessments that failed to incorporate HIV status among covariates [16-22]. Despite it not being implicated, higher rates of modern contraceptive methods use particularly consistent condom use had been reported among HIV-positive women [42, 43]. Regardless, contraception expansion efforts are crucial in Cameroon as those in the lower socioeconomic ranks would benefit more [44] from improved availability, accessibility, or affordability.

This study not only contributes in terms of uncovering disparities to inform family planning policy formulation but could benefit local-level monitoring of progress toward global benchmarks such as the proposed SDG for attainment of ≥ 75% demand satisfied with modern methods by 2030 [6]. However, it is not without limitations. Firstly, this relates to the uncertainty associated with data for smaller areas (i.e., socioeconomic categories per region) as Cameroon-DHS data is powered at the region level [36]. Extra levels of hierarchy representing the socioeconomic categories, regions, and time were thus introduced to allow for greater pooling of information across the smaller populations [45]. However, the findings should still be interpreted

with caution. Furthermore, due to other data limitations, indirect impacts of important processes such as family planning policy changes were not factored into the model. While such processes could be reflected in incorporated variables like the HDIs and/or captured in the data collected at each survey point, we aimed to generate estimates mainly based on current trends. Regarding the determinants analysis, the causal links between examined factors relating to the use- and non-use of modern contraceptives are difficult to establish due to the study's cross-sectional nature. Additionally, this study excludes unmarried women of reproductive age even though they make up increasing proportions of users of family planning services [46]. Given the discrepancies in definitions of the demand for family planning between the two groups [28, 46], we aim to conduct a separate assessment for the unmarried population.

Conclusion

Wide urban-rural, wealth-, and education-based disparities remain in the coverage of modern contraceptive methods of family planning among married women of reproductive age across the regions of Cameroon. At most, ≥ 75% demand satisfied with modern methods could be attained in only one-third of rural areas, one-sixth of the two poorest quintiles, and one-quarter of two bottom educational categories by 2030. The findings presented should inform local-level family planning policies in terms of what groups (i.e., rural, less educated, and poorer women) to prioritise in each region. Meanwhile, the determinants analysis suggests the factoring of age group, number of living children, level of education, wealth status, and religion when designing family planning interventions. Future studies examining locality-specific influences on the uptake of family planning services and commodities could further inform programmes at the regional level.

Supporting information

S1 Table. Characteristics of data used to assess family planning indicators by area of residence, wealth, and educational status across regions of Cameroon. DHS=Demographic and Health Survey; mCPR=modern contraceptive prevalence; mUNFP=unmet need for modern methods; mDSFP=demand satisfied with modern methods. Sample= women aged 15 to 49 years who are married or in a union

S2 Table. Definitions of the coverage for family planning indicators. Married women includes those who are married or in a union. Total number of women with a demand for family

planning (modern contraceptive prevalence + traditional contraceptive prevalence + unmet need). Modern contraceptive methods include sterilisations, oral contraceptive pills, intrauterine devices, injectables, implants, condoms, and lactational amenorrhea method, standard days method, emergency contraception and vaginal barrier methods.

S3 Table. Use of, unmet need, and demand satisfied for modern contraceptive methods by rural and urban residence across regions of Cameroon, 2015 and 2030. Estimates are in % (95% Credible Interval)

S4 Table. Use of, unmet need, and demand satisfied for modern contraceptive methods by wealth quintile across regions of Cameroon, 2015 and 2030. Estimates are in % (95% Credible Interval); Q1=poorest, Q2=poorer, Q3=middle, Q4=richer, Q5=richest.

S5 Table. Magnitude of socio-economic inequalities in the use of, unmet need, and demand satisfied for modern contraceptive methods across regions of Cameroon, 2015 and 2030. %p= percentage points.

S6 Table. Use of, unmet need, and demand satisfied for modern contraceptive methods by level of education across regions of Cameroon, 2015 and 2030. Estimates are in % (95% Credible Interval); E1=none, E2=primary, E3=secondary, E4=higher levels of educational attainment.

S7 Table. Determinants of use of and unmet need for modern methods of family planning in Cameroon, DHS 2018. Modern methods=Modern contraceptive methods; Media exposure represents a combination of variables for frequency of reading newspapers or magazines, listening to radio, watching television, and use of internet; CrI=Credible Interval.

S8 Table. Differences in posterior means of main and altered (no predictor) models by area of residence. HDI=Human Development Index.

S9 Table. Differences in posterior means of main and altered (weak priors) models by area of residence

S10 Table. Differences in posterior means of main and altered (no predictor) models by wealth quintile. HDI=Human Development Index; Q1=poorest, Q2=poorer, Q3=middle, Q4=richer, and Q5=richest.

S11 Table. Differences in posterior means of main and altered (weak priors) models by wealth quintile. Q1=poorest, Q2=poorer, Q3=middle, Q4=richer, and Q5=richest.

S12 Table. Differences in posterior means of main and altered (no predictor) models by education level. HDI=Human Development Index; E1=none, E2=primary, E3=secondary, E4=higher.

S13 Table. Differences in posterior means of main and altered (weak priors) models by education level. E1=none, E2=primary, E3=secondary, E4=higher.

S1 Appendix. S-Methods: Bayesian analysis.

Author Contributions

Conceptualization: Raïssa Shiyghan Nsashiyi, Md Mizanur Rahman, Lawrence Monah Ndam, Masahiro Hashizume.

Formal analysis: Raïssa Shiyghan Nsashiyi, Lawrence Monah Ndam.

Methodology: Raïssa Shiyghan Nsashiyi, Md Mizanur Rahman.

Supervision: Md Mizanur Rahman, Lawrence Monah Ndam, Masahiro Hashizume.

Writing – original draft: Raïssa Shiyghan Nsashiyi.

Writing – review & editing: Raïssa Shiyghan Nsashiyi, Md Mizanur Rahman, Lawrence Monah Ndam, Masahiro Hashizume.

References

1. UN General Assembly. Transforming our world: the 2030 Agenda for Sustainable Development 2015. Available from: https://www.un.org/en/development/desa/population/migration/generalassembly/docs/globalcompact/A_RES_70_1_E.pdf.
2. Ogundele OJ, Pavlova M, Groot W. Examining trends in inequality in the use of reproductive health care services in Ghana and Nigeria. BMC pregnancy and childbirth. 2018;18(1):492. Epub 2018/12/14. doi: 10.1186/s12884-018-2102-9. PubMed PMID: 30545328; PubMed Central PMCID: PMCPMC6293518.
3. FP2020. Family Planning 2020 (FP2020) is a global movement that supports the rights of women and girls to decide – freely and for themselves – whether, when, and how many children they want to have. 2020 [cited 2020 15 Aug]. Available from: http://www.familyplanning2020.org/about-us.
4. Alkema L, Kantorova V, Menozzi C, Biddlecom A. National, regional, and global rates and trends in contraceptive prevalence and unmet need for family planning between 1990 and 2015: a systematic and comprehensive analysis. Lancet. 2013;381(9878):1642-52. Epub 2013/03/16. doi: 10.1016/S0140-6736(12)62204-1. PubMed PMID: 23489750.
5. Cahill N, Sonneveldt E, Stover J, Weinberger M, Williamson J, Wei C, et al. Modern contraceptive use, unmet need, and demand satisfied among women of reproductive age who are married or in a union in the focus countries of the Family Planning 2020 initiative: a systematic analysis using the Family Planning Estimation Tool. Lancet. 2018;391(10123):870-82. Epub 2017/12/09. doi: 10.1016/s0140-6736(17)33104-5. PubMed PMID: 29217374; PubMed Central PMCID: PMCPMC5854461.
6. Choi Y, Short Fabic M. Monitoring Progress in Equality for the Sustainable Development Goals: A Case Study of Meeting Demand for Family Planning. Glob Health Sci Pract. 2018;6(2):390-401. doi: 10.9745/GHSP-D-18-00012. PubMed PMID: 29959277.
7. Chola L, McGee S, Tugendhaft A, Buchmann E, Hofman K. Scaling Up Family Planning to Reduce Maternal and Child Mortality: The Potential Costs and Benefits of Modern Contraceptive Use in South Africa. PloS one. 2015;10(6):e0130077. Epub 2015/06/16. doi: 10.1371/journal.pone.0130077. PubMed PMID: 26076482; PubMed Central PMCID: PMCPMC4468244.

8. Hounton S, Barros AJ, Amouzou A, Shiferaw S, Maïga A, Akinyemi A, et al. Patterns and trends of contraceptive use among sexually active adolescents in Burkina Faso, Ethiopia, and Nigeria: evidence from cross-sectional studies. Global health action. 2015;8:29737. Epub 2015/11/13. doi: 10.3402/gha.v8.29737. PubMed PMID: 26562143; PubMed Central PMCID: PMCPMC4642360.
9. Ewerling F, Victora CG, Raj A, Coll CVN, Hellwig F, Barros AJD. Demand for family planning satisfied with modern methods among sexually active women in low- and middle-income countries: who is lagging behind? Reproductive health. 2018;15(1):42. Epub 2018/03/08. doi: 10.1186/s12978-018-0483-x. PubMed PMID: 29510682; PubMed Central PMCID: PMCPMC5840731.
10. FP2020. Cameroon, Commitment Maker Since 2014. 2020 [cited 2020 15 Aug]. Available from: https://www.familyplanning2020.org/cameroon.
11. Gichangi P, Agwanda A, Thiongo M, Waithaka M, Tsui A, Radloff S, et al. Assessing (in) equalities in contraceptives use and family planning demand satisfied with modern contraceptives in Kenya. Research Square; 2020.
12. Budu E, Dadzie LK, Salihu T, Ahinkorah BO, Ameyaw EK, Aboagye RG, et al. Socioeconomic inequalities in modern contraceptive use among women in Benin: a decomposition analysis. BMC women's health. 2023;23(1):444. Epub 2023/08/24. doi: 10.1186/s12905-023-02601-y. PubMed PMID: 37612594; PubMed Central PMCID: PMCPMC10463475.
13. Mensah F, Okyere J, Azure SA, Budu E, Ameyaw EK, Seidu AA, et al. Age, geographical and socio-economic related inequalities in contraceptive prevalence: evidence from the 1993-2014 Ghana Demographic and Health Surveys. Contraception and reproductive medicine. 2023;8(1):20. Epub 2023/02/09. doi: 10.1186/s40834-022-00194-9. PubMed PMID: 36750918; PubMed Central PMCID: PMCPMC9903545.
14. Kanwal Aslam S, Zaheer S, Qureshi MS, Aslam SN, Shafique K. Socio-Economic Disparities in Use of Family Planning Methods among Pakistani Women: Findings from Pakistan Demographic and Health Surveys. PloS one. 2016;11(4):e0153313. Epub 2016/04/08. doi: 10.1371/journal.pone.0153313. PubMed PMID: 27055164; PubMed Central PMCID: PMCPMC4824490.
15. Nsashiyi RS, Rahman MM, Ndam LM, Hashizume M. Exploiting the Bayesian approach to derive counts of married women of reproductive age across Cameroon for healthcare planning, 2000-2030. Sci Rep. 2022;12(1):18075. Epub 2022/10/28. doi: 10.1038/s41598-022-23089-w. PubMed PMID: 36302837; PubMed Central PMCID: PMCPMC9613669.
16. Lasong J, Zhang Y, Gebremedhin SA, Opoku S, Abaidoo CS, Mkandawire T, et al. Determinants of modern contraceptive use among married women of reproductive age: a cross-sectional study in rural Zambia. BMJ open. 2020;10(3):e030980. Epub 2020/04/03. doi: 10.1136/bmjopen-2019-030980. PubMed PMID: 32234737; PubMed Central PMCID: PMCPMC7170561.
17. Gafar A, Suza DE, Efendi F, Has EMM, Pramono AP, Susanti IA. Determinants of contraceptive use among married women in Indonesia. F1000Research. 2020;9:193. Epub 2020/04/10. doi: 10.12688/f1000research.22482.1. PubMed PMID: 32269768; PubMed Central PMCID: PMCPMC7137393.
18. Nzokirishaka A, Itua I. Determinants of unmet need for family planning among married women of reproductive age in Burundi: a cross-sectional study. Contraception and reproductive medicine. 2018;3:11. Epub 2018/06/29. doi: 10.1186/s40834-018-0062-0. PubMed PMID: 29951222; PubMed Central PMCID: PMCPMC6011199 published maps and institutional applications.The authors declare that they have no competing interests.Springer Nature remains neutral with regard to jurisdictional claims in published maps and institutional affiliations.
19. Nyarko SH. Spatial variations and socioeconomic determinants of modern contraceptive use in Ghana: A Bayesian multilevel analysis. PloS one. 2020;15(3):e0230139. Epub 2020/03/11. doi: 10.1371/journal.pone.0230139. PubMed PMID: 32155217; PubMed Central PMCID: PMCPMC7064218.

20. Asif MF, Pervaiz Z. Socio-demographic determinants of unmet need for family planning among married women in Pakistan. BMC public health. 2019;19(1):1226. Epub 2019/09/07. doi: 10.1186/s12889-019-7487-5. PubMed PMID: 31488094; PubMed Central PMCID: PMCPMC6727575.

21. Ajmal S, Idris A, Ajmal B. Factors affecting contraceptive use and unmet need among currently married women in Afghanistan: further analysis of the 2015 Afghanistan Demographic and Health Survey. Journal of Global Health Reports. 2018;2:e2018037.

22. Yussuf MH, Elewonibi BR, Rwabilimbo MM, Mboya IB, Mahande MJ. Trends and predictors of changes in modern contraceptive use among women aged 15-49 years in Tanzania from 2004-2016: Evidence from Tanzania Demographic and Health Surveys. PloS one. 2020;15(6):e0234980. Epub 2020/07/01. doi: 10.1371/journal.pone.0234980. PubMed PMID: 32598371; PubMed Central PMCID: PMCPMC7323946.

23. Frank TD, Carter A, Jahagirdar D, Biehl MH, Douwes-Schultz D, Larson SL, et al. Global, regional, and national incidence, prevalence, and mortality of HIV, 1980-2017, and forecasts to 2030, for 195 countries and territories: a systematic analysis for the Global Burden of Diseases, Injuries, and Risk Factors Study 2017. The lancet HIV. 2019;6(12):e831-e59. Epub 2019/08/24. doi: 10.1016/s2352-3018(19)30196-1. PubMed PMID: 31439534; PubMed Central PMCID: PMCPMC6934077.

24. Holmes KK, Levine R, Weaver M. Effectiveness of condoms in preventing sexually transmitted infections. Bulletin of the World Health Organization. 2004;82(6):454-61. Epub 2004/09/11. PubMed PMID: 15356939; PubMed Central PMCID: PMCPMC2622864.

25. Stover J, Teng Y. The impact of condom use on the HIV epidemic: Gates Open Res. 2022 Feb 11;5:91. doi: 10.12688/gatesopenres.13278.2. eCollection 2021.; 2021.

26. Habte D, Namasasu J. Family planning use among women living with HIV: knowing HIV positive status helps - results from a national survey. Reproductive health. 2015;12:41. Epub 2015/05/11. doi: 10.1186/s12978-015-0035-6. PubMed PMID: 25958383; PubMed Central PMCID: PMCPMC4435559.

27. New JR, Cahill N, Stover J, Gupta YP, Alkema L. Levels and trends in contraceptive prevalence, unmet need, and demand for family planning for 29 states and union territories in India: a modelling study using the Family Planning Estimation Tool. The Lancet Global health. 2017;5(3):e350-e8. Epub 2017/02/15. doi: 10.1016/s2214-109x(17)30033-5. PubMed PMID: 28193400.

28. Bradley SEK, Croft TN, Fishel JD, Westoff CF. Revising unmet need for family planning. Calverton, Maryland, USA: ICF International, 2012.

29. Ahmed S, Choi Y, Rimon JG, Alzouma S, Gichangi P, Guiella G, et al. Trends in contraceptive prevalence rates in sub-Saharan Africa since the 2012 London Summit on Family Planning: results from repeated cross-sectional surveys. The Lancet Global health. 2019;7(7):e904-e11. Epub 2019/05/22. doi: 10.1016/s2214-109x(19)30200-1. PubMed PMID: 31109881; PubMed Central PMCID: PMCPMC6560024.

30. Global Data Lab. Subnational Human Development Index (4.0): Institute for Management Research Radboud University; 2021. Available from: https://globaldatalab.org/shdi/shdi/CMR/?levels=1%2B4&interpolation=0&extrapolation=0&nearest_real=0.

31. UNDP. UNITED NATIONS DEVELOPMENT PROGRAMME: Human Development Reports. Human Development Index (HDI). 2021 [cited 2021 8 July]. Available from: http://hdr.undp.org/en/content/human-development-index-hdi.

32. Komasawa M, Yuasa M, Shirayama Y, Sato M, Komasawa Y, Alouri M. Demand for family planning satisfied with modern methods and its associated factors among married women of reproductive age in rural Jordan: A cross-sectional study. PloS one. 2020;15(3):e0230421. Epub 2020/03/19. doi: 10.1371/journal.pone.0230421. PubMed PMID: 32187224; PubMed Central PMCID: PMCPMC7080244.

33. Gelman A, Carlin BJ, Stern SH, Dunson BD, Vehtari A, Rubin BD. Bayesian Data Analysis Third Edition: Boca Raton. FL: CRC Press; 2020.

34. Gelman A, Rubin DB. Inference from Iterative Simulation Using Multiple Sequences. Statist Sci. 1992;7(4):457-72. doi: 10.1214/ss/1177011136.
35. Boerma T, AbouZahr C, Evans D, Evans T. Monitoring intervention coverage in the context of universal health coverage. PLoS medicine. 2014;11(9):e1001728. Epub 2014/09/23. doi: 10.1371/journal.pmed.1001728. PubMed PMID: 25243586; PubMed Central PMCID: PMCPMC4171108.
36. Institut National de la Statistique (INS) et ICF International. Enquête Démographique et de Santé du Cameroun 2018. Yaoundé, Cameroun et Rockville, Maryland, USA : INS et ICF [Producers] ICF [Distributor], . 2020.
37. World Bank. Cameroon - Public Expenditure Review: Aligning Public Expenditures with the Goals of Vision 2035 (English). Washington, D.C. : World Bank Group, 2018.
38. Sandie AB, Tchatchueng Mbougua JB, Nlend AEN, Thiam S, Nono BF, Fall NA, et al. Hot-spots of HIV infection in Cameroon: a spatial analysis based on Demographic and Health Surveys data. BMC infectious diseases. 2022;22(1):334. Epub 2022/04/06. doi: 10.1186/s12879-022-07306-5. PubMed PMID: 35379192; PubMed Central PMCID: PMCPMC8981942.
39. U. Hanemann (Ed.). The Study Groups and Literacy Programme, Cameroon. UNESCO Institute for Lifelong Learning: National Association of Cameroonian Language Committees; 2017 [cited 2021 8 July]. Available from: https://uil.unesco.org/case-study/effective-practices-database-litbase-0/study-groups-and-literacy-programme-cameroon.
40. Ngala D. Quality vs. Quantity: The Private University Institute Boom in Buea, Cameroon 2023 [cited 2023 December 9]. Available from: https://medium.com/@diranengala/quality-vs-quantity-the-private-university-institute-boom-in-buea-cameroon-cbc92fb563f2.
41. Alkema L, Kantorova V, Menozzi C, Biddlecom A. National, regional, and global rates and trends in contraceptive prevalence and unmet need for family planning between 1990 and 2015: a systematic and comprehensive analysis. Lancet. 2013;381(9878 (suppl p13)):1642-52. Epub 2013/03/16. doi: 10.1016/S0140-6736(12)62204-1. PubMed PMID: 23489750.
42. Mersha AG, Erku DA, Belachew SA, Ayele AA, Gebresillassie BM, Abegaz TM. Contraceptive use among HIV-positive and negative women: implication to end unintended pregnancy. Contraception and reproductive medicine. 2019;4:3. Epub 2019/02/21. doi: 10.1186/s40834-019-0084-2. PubMed PMID: 30783537; PubMed Central PMCID: PMCPMC6376717.
43. Kaida A, Laher F, Strathdee SA, Money D, Janssen PA, Hogg RS, et al. Contraceptive use and method preference among women in Soweto, South Africa: the influence of expanding access to HIV care and treatment services. PloS one. 2010;5(11):e13868. Epub 2010/11/17. doi: 10.1371/journal.pone.0013868. PubMed PMID: 21079770; PubMed Central PMCID: PMCPMC2974641.
44. Vlassoff M, Jerman J, Beninguisse G, Kamgaing F, Zinvi-Dossou F. Benefits of meeting the contraceptive needs of Cameroonian women. Issues in brief (Alan Guttmacher Institute). 2014;(1):1-13. Epub 2014/09/10. PubMed PMID: 25199220.
45. Susmann H, Sakrejda K, Wei C, Alkema L. Small area estimation of modern contraceptive use in Kenya using the Family Planning Estimation Tool. Extended abstract prepared for PAA 2019. 2019.
46. Kantorová V, Wheldon MC, Ueffing P, Dasgupta ANZ. Estimating progress towards meeting women's contraceptive needs in 185 countries: A Bayesian hierarchical modelling study. PLoS medicine. 2020;17(2):e1003026. Epub 2020/02/19. doi: 10.1371/journal.pmed.1003026. PubMed PMID: 32069289; PubMed Central PMCID: PMCPMC7028249.

Figure 1

Click here to access/download;Figure;Fig1.tiff.tiff

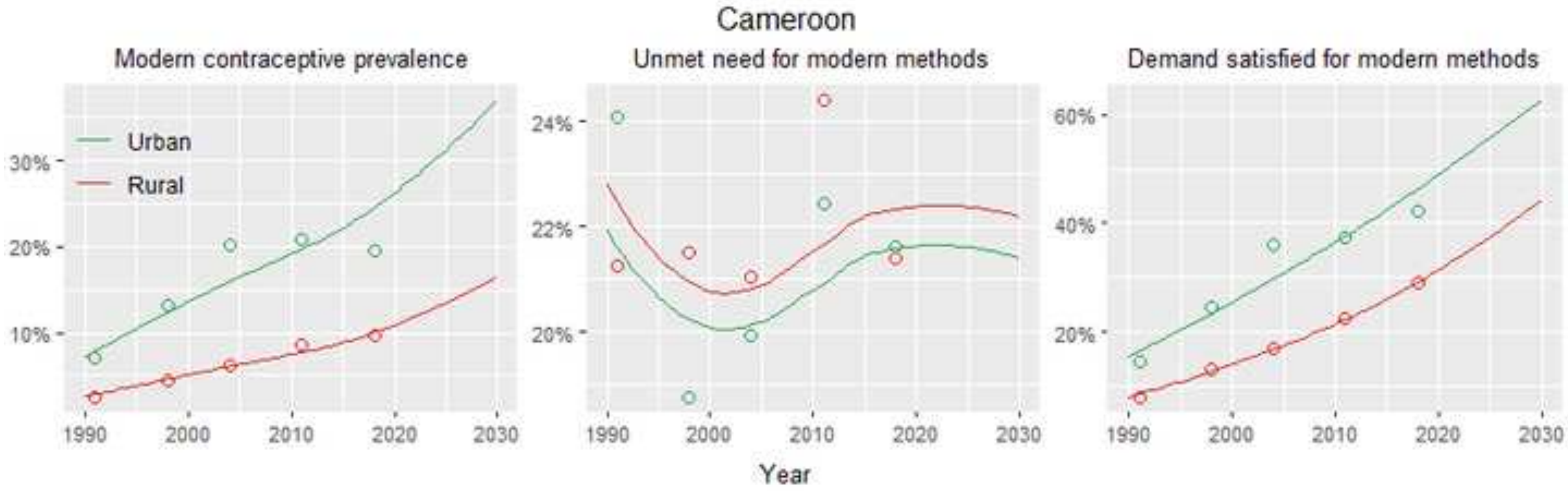

Figure 2 Click here to access/download;Figure;Fig2.tiff.tiff

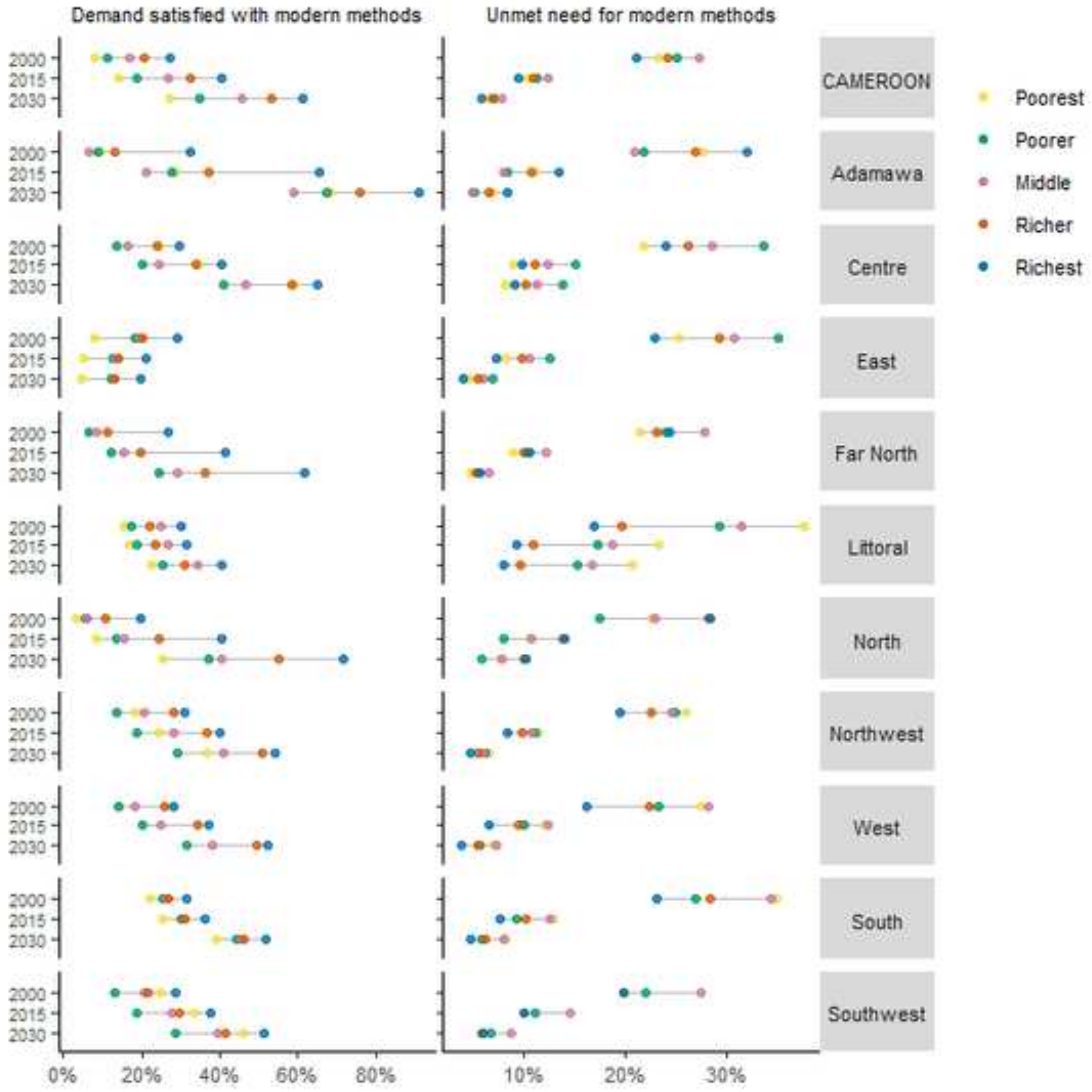

Figure 3 Click here to access/download;Figure;Fig3.tiff.tiff

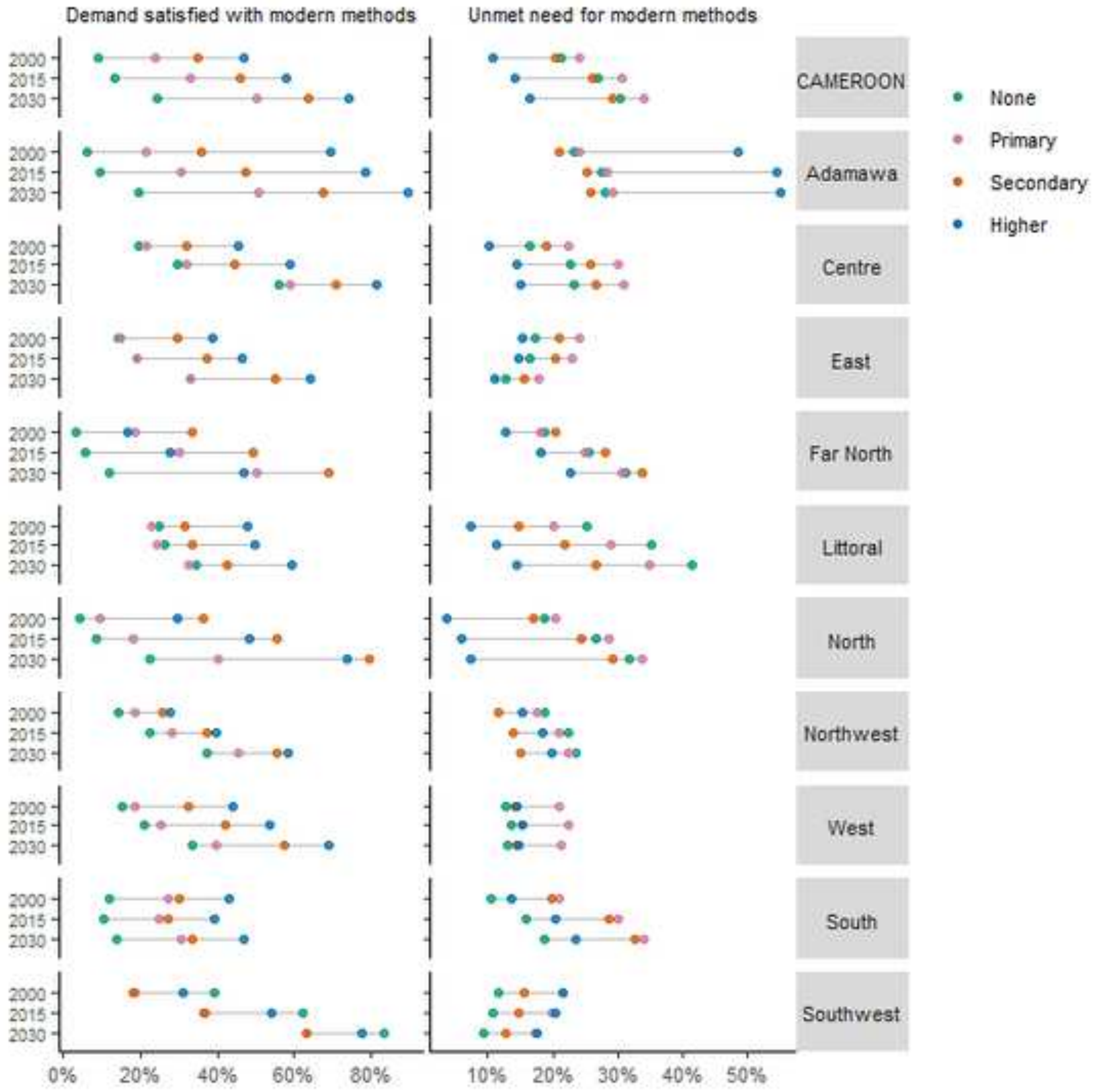

Click here to access/download
Supporting Information
S1_Appendix.docx

Click here to access/download
Supporting Information
S1_Table.docx

S2_Table

Click here to access/download
Supporting Information
S2_Table.docx

Click here to access/download
Supporting Information
S3_Table.docx

Click here to access/download
Supporting Information
S4_Table.docx

Click here to access/download
Supporting Information
S5_Table.docx

Click here to access/download
Supporting Information
S6_Table.docx

Click here to access/download
Supporting Information
S7_Table.docx

Click here to access/download
Supporting Information
S8_Table.docx

Click here to access/download
Supporting Information
S9_Table.docx

Click here to access/download
Supporting Information
S10_Table.docx

Click here to access/download
Supporting Information
S11_Table.docx

Click here to access/download
Supporting Information
S12_Table.docx

S13_Table

Click here to access/download
Supporting Information
S13_Table.docx
