# Supplementary material for: A subnational socioeconomic assessment of family planning levels, projections, and disparities among married women of reproductive age in Cameroon
Source: PLoS One. 2025 Feb 14;20(2):e0318650. doi: 10.1371/journal.pone.0318650 (PMC11828404; doi:10.1371/journal.pone.0318650)
Supplement: Attachment — Submitted filename: Response to Reviewers.pdf [file pone.0318650.s016.pdf]

Response to Reviewers

==

Journal Requirements:

1. When submitting your revision, we need you to address these additional requirements.

Please ensure that your manuscript meets PLOS ONE's style requirements, including those for file naming. The PLOS ONE style templates can be found at https://journals.plos.org/plosone/s/file?id=wjVg/PLOSOne_formatting_sample_main_body.pdf and https://journals.plos.org/plosone/s/file?id=ba62/PLOSOne_formatting_sample_title_authors_affiliations.pdf

Response to point 1: Thank you. The manuscript has been revised in line with the PLOS ONE's style requirements provided in the linked documents.

2. Please amend either the title on the online submission form (via Edit Submission) or the title in the manuscript so that they are identical.

Response to point 2: Thank you. The title on the online submission form has been amended to be identical to the title in the manuscript.

3. Please review your reference list to ensure that it is complete and correct. If you have cited papers that have been retracted, please include the rationale for doing so in the manuscript text, or remove these references and replace them with relevant current references. Any changes to the reference list should be mentioned in the rebuttal letter that accompanies your revised manuscript. If you need to cite a retracted article, indicate the article's retracted status in the References list and also include a citation and full reference for the retraction notice.

Response to point 3: Thank you. The reference list has been crosschecked and updated according to the specifications mentioned above.

Three references have been added to the reference list to support revisions made, including some in line with some of the reviewers' comments. These include;

In-text Line(s)	Citation #	Reference	Rationale
356–359	[41]	41. Sultan S. The Effects of Education, Poverty, and Resources on Family Planning in Developing	To support revision made in response to

		Countries. Clinics in Mother and Child Health. 2018;15. doi: 10.4172/2090-7214.1000289.	Reviewer #1's Comment 6 below
394–397	[44]	44. Nimani TD, Tadese ZB, Tadese EE, et al. Trend, geographical distribution, and determinants of modern contraceptive use among married reproductive-age women, based on the 2000, 2005, 2011, and 2016 Ethiopian demographic and health survey. BMC women's health 2023;23(1):629. doi: 10.1186/s12905-023-02789-z	To support revision made in response to Reviewer #1's Comment 8 below
70–71<br><br>349–352	[10]	10. Nsashiyi RS, Rahman MM, Ndam LM, et al. Contraceptive use, unmet need, and demand satisfied for family planning across Cameroon: a subnational study including indirect effects of COVID-19 and armed conflict on projections. BMC Global and Public Health 2024;2(1):40. doi: 10.1186/s44263-024-00071-4	To insert a more relevant recently published article that aligns with the statements made

==

Reviewer #1: Please find my comments below:

Comment 1: First of all, it is important to know which definition of unmet need used in the manuscript.

Response 1: Thank you for your feedback.

We defined "Unmet need for modern methods is the percentage of women who are not currently using any method of contraception to prevent pregnancy but want to space or limit childbearing." (Lines 105–107)

The following statement has been added for clarity "...This indicator also includes women using traditional family planning methods, as they are considered to have an unmet need for more effective modern contraceptive methods. (Lines 107–109)

Our definition was drawn from the DHS revised definition of unmet need (Bradley et al. (2012) [Citation number 29]: https://dhsprogram.com/pubs/pdf/AS25/AS25[12June2012].pdf), which also includes women who are using a traditional method of family planning.

Comment 2: Also, It is important to provide information on average marriage age, especially for women.

It is important to provide information on average marriage age, especially for women.

Response 2: Thank you. Average marriage age has been added to the descriptive results (i.e., in Line 266–267)
Comment 3: In the line 96, the writing of the figure 1,5426 is not correct

Response 3: Thank you. The figure has been corrected to “15,426” (Line 99)

Comment 4: In the lines 102 and 103, the method can be used by woman or her husband/ partner

Response 4: Thank you. The definition has been revised to include husbands/partners (Line 104).

Comment 5: There are something which not follow the main trend. Their should be considered in the discussion.

There were some exceptions which was not addressed in the discussion so far.

Response 5: Thank you for highlighting this. The unexpected findings/exceptions have been discussed thus (Lines 402–409);

“Notably, the study shows unexpected lower rates of modern contraceptive use among women with higher compared to secondary education, in the richer compared to middle wealth quintile, and parity ≥6 compared to parity 4–6. Recognising the potential influence of study limitations, these findings suggest that women with higher educational attainment or from wealthier households may prioritise career or personal development over immediate reproductive needs. Also, women with more children may experience "fertility inertia" or less likely to seek contraception after achieving their desired family size.”

Comment 6: Regarding the line 336 and next sentences It is very important to prioritize the poorer and less educated people. But simultaneously, country should develop programs for increasing the literacy level as a standing agenda and a cornerstone for development issues. The social policy and social security relevant organization need to develop the relevant programs.

Response 6: Thank you for your suggestion. The statements have been revised (Lines 356–359) thus;

“Simultaneously, the country should focus on increasing literacy and reducing poverty through its development initiatives as well as collaborative efforts with social services organisations, to enhance understanding and empower decision-making in family planning, particularly for these disadvantaged groups [41].”

Comment 7: In the line 354 what is the meaning of positive trend. Does it mean the gap decrease or the figure increase. For me it is vague.

Response 7: Thank you for pointing this out. ‘Positive’ and ‘Negative’ have been replaced with ‘Increasing’ and ‘Decreasing’. The sentence has been revised (Lines 376–378) thus;

“More unclear are the education-related patterns of unmet need for modern methods, as trends across categories showed increasing levels in most regions (6 out of 10), and decreasing or steady in the rest.

Comment 8: In the line 366 In the text, more than 20 years old is a predictor for increasing contraceptive use. As a usual, with increasing the age and going to the older generation, contraceptive use may decrease. It is necessary to have a short explanation on it.

Response 8: Thank you. We have reinforced the discussions with your recommendation, thus (Lines 394–397);

“Estimates showing that women aged 20 to 29 and 30 to 39 are at least twice more likely to use modern contraceptives compared to 15 to 19-year-olds aligns with literature, which indicates that contraceptive use trends increase with age until they begin to decline in older generations [23, 44].”

Comment 9: In the line 376 and next sentences, uncovering disparities and its reasons will help policymakers to develop a plan toward SDG goals. It will show that different region in the country needs different approaches and intervention.

Response 9: Thank you very much. Your suggestion has been incorporated, thus (Lines 422–424);

“The findings should enable Cameroon to adopt varied approaches for family planning implementation and expansion, tailored to the disparities identified in each region.”

Comment 10: In the conclusion, it is necessary to think about multi-sectoral

cooperation for improvement of literacy rate, social support schemes, provision of services with subsidy, improvement of access ...

Response 10: The conclusion has been reinforced with your suggestion, thus (Lines 448–451);

“To effectively address these disparities, multi-sectoral cooperation is essential. This includes initiatives aimed at improving literacy rates, implementing social support schemes, providing subsidised services, and enhancing access to family planning resources.”

==

Reviewer #2:

Comment: There is no substantial comment to be forwarded, How ever, I recommend the abstract to be rewritten to give a brief and explained background information regarding the topic "interest socioeconomic assessment of family planning levels". Additionally, the conclusion section should be revised to make it focused on the major results.

Response: Thank you for your valuable feedback. The abstract background has been revised to demonstrate our "interest socioeconomic assessment of family planning levels" (Lines 27–31).

==
