# Supplementary material for: A subnational socioeconomic assessment of family planning levels, projections, and disparities among married women of reproductive age in Cameroon
Source: PLoS One. 2025 Feb 14;20(2):e0318650. doi: 10.1371/journal.pone.0318650 (PMC11828404; doi:10.1371/journal.pone.0318650)
Supplement: Attachment — Submitted filename: Response_to_Reviewers_auresp_2.pdf [file pone.0318650.s017.pdf]

Response to Reviewers

==

Journal Requirements:

Please review your reference list to ensure that it is complete and correct. If you have cited papers that have been retracted, please include the rationale for doing so in the manuscript text, or remove these references and replace them with relevant current references. Any changes to the reference list should be mentioned in the rebuttal letter that accompanies your revised manuscript. If you need to cite a retracted article, indicate the article's retracted status in the References list and also include a citation and full reference for the retraction notice.

Response:

Thank you for your guidance regarding the reference list. We have thoroughly cross-checked the reference list to ensure that it is complete and accurate. No new references were added; however, we have made several edits for consistency, including the addition of punctuation marks (full stops '.'), proper spacing, and the inclusion of 'https://' before the digital object identifier (DOI) for each article.

We confirm that there are no retracted papers in our reference list.

==

Reviewer #1: Thanks. The comments were mostly addressed. I couldn't find the answer to one comment which was related to the average of marriage among the community. You already mentioned it was addressed but, I couldn't find it.

Thank you for your comment.

We have included the mean age of the respondents (i.e., married women of reproductive age) in the descriptive results, as the average age of marriage among the community was not captured in the dataset we utilized. Additionally, we were unable to locate reliable or scientifically sound sources that provide data on the average age of marriage in Cameroon.

To ensure clarity, we have explicitly stated the average age of the respondents in the manuscript. Specifically, Lines 266-268 have been revised to read:

"The descriptive results (Table 3) indicate that among the 1,513 respondents, the average age was 30.8 years (SD ±7.9), with a considerable proportion falling within the 20–29 (38.6%) and 30–39 (37.9%) age groups, while only 7.7% were aged 15–19."

We appreciate your valuable feedback and hope this revision addresses your concerns.

==
